# Inventory of telomerase components in human cells reveals multiple subpopulations of hTR and hTERT

**DOI:** 10.1093/nar/gku560

**Published:** 2014-07-02

**Authors:** Linghe Xi, Thomas R. Cech

**Affiliations:** 1 University of Colorado BioFrontiers Institute, Boulder, CO 80303, USA; 2 Department of Molecular, Cellular and Developmental Biology, University of Colorado, Boulder, CO 80309, USA; 3 Howard Hughes Medical Institute and Department of Chemistry and Biochemistry, University of Colorado, Boulder, CO 80303, USA

## Abstract

Telomerase is the ribonucleoprotein (RNP) enzyme that elongates telomeric DNA to compensate for the attrition occurring during each cycle of DNA replication. Knowing the levels of telomerase in continuously dividing cells is important for understanding how much telomerase is required for cell immortality. In this study, we measured the endogenous levels of the human telomerase RNP and its two key components, human telomerase RNA (hTR) and human telomerase reverse transcriptase (hTERT). We estimate ∼240 telomerase monomers per cell for HEK 293T and HeLa, a number similar to that of telomeres in late S phase. The subunits were in excess of RNPs (e.g. ∼1150 hTR and ∼500 hTERT molecules per HeLa cell), suggesting the existence of unassembled components. This hypothesis was tested by overexpressing individual subunits, which increased total telomerase activity as measured by the direct enzyme assay. Thus, there are subpopulations of both hTR and hTERT not assembled into telomerase but capable of being recruited. We also determined the specific activity of endogenous telomerase and of overexpressed super-telomerase both to be ∼60 nt incorporated per telomerase per minute, with *K*_m_(dGTP) ∼17 μM, indicating super-telomerase is as catalytically active as endogenous telomerase and is thus a good model for biochemical studies.

## INTRODUCTION

Telomeres, the complex structures present at the ends of linear eukaryotic chromosomes, have essential roles in maintaining genome stability and controlling cell proliferation (1). The DNA in telomeres consists of a highly repetitive sequence, which is covered by specific telomere-associated proteins (1). This repetitive DNA sequence is synthesized by a specialized reverse transcriptase enzyme, telomerase. In mammals, the activity of telomerase is required by stem cells and the germline, and loss of telomerase activity in these cells results in decreased proliferative capacity (2,3). However, improper activation of telomerase in somatic cells contributes to tumorigenesis (4). Being tightly linked to significant health concerns like aging and cancer, telomerase has been a focus of research for two decades.

From a biochemical point of view, telomerase has attracted attention because it is a ribonucleoprotein (RNP) enzyme. An RNA component, telomerase RNA (TR), and a protein component, telomerase reverse transcriptase (TERT), are assembled into a core complex that catalyzes the synthesis of telomeric DNA (5–7). These two components are conserved in all organisms expressing telomerase. Accessory proteins, which vary amongst different species, associate with this core complex and serve to orchestrate its biogenesis and regulate its activity (1). In human, the most clearly established accessory proteins are dyskerin, which stabilizes TR in the nucleus (1,8,9), and telomerase Cajal body protein 1 (TCAB1), which localizes telomerase to Cajal bodies (1,10,11).

It has been extremely valuable in the telomere/telomerase field to know how many complexes are present per cell, not so much because exact numbers are important, but because accurate quantitative data can lead to conceptual insights. For example, careful measurements of the abundance of telomere-associated shelterin complex subunits in human cells allowed Takai *et* *al*. (12) to conclude that there was enough telomeric repeat-binding factor 1 (TRF1) and telomeric repeat-binding factor 2 (TRF2) to coat all the telomeric dsDNA in cells, that there was more than enough protection of telomeres 1 (POT1) and TPP1 to coat the ssDNA at the very ends of chromosomes, and that there must be shelterin subcomplexes in addition to the full six-protein complex. As another example, measurements of the abundance of TR in budding yeast allowed Mozdy and Cech (13) to conclude that there were fewer telomerase complexes than telomeres in the late S phase of both haploid and diploid cells. Thus, if telomerase is tethered to the telomere, not all telomeres can be replicated in every cell cycle. Furthermore, limiting telomerase predicts haploinsufficiency, which was experimentally confirmed.

Given the value of knowing the number of telomerase complexes per human cell, a number of research groups have reported measurements of hTR levels. However, the reported values are not in concordance. Yi *et* *al*. (14) measured ∼60 000 hTR molecules per cell in both HeLa and HEK 293 cell lines, which, if all assembled into active telomerase, would be in great excess over telomeres. On the other hand, Cao *et* *al*. (15) measured ∼120 molecules of hTR per HEK 293 cell, in which case there would be fewer telomerase complexes than telomeres (given that HEK 293 cells are hypotriploid with a modal chromosome number of 64, so there are 256 telomeres after S phase (16)).

Furthermore, because hTR appears to be present in many types of human cells regardless of their telomerase enzymatic activity, whereas the presence of hTERT correlates with enzymatic activity (6,7,17–19), there is a general view that hTERT is the limiting component for telomerase assembly. However, telomerase activity in human T lymphocytes has been reported to relate to hTR levels but not hTERT protein levels (20,21). Clearly quantitative measurements of the hTERT protein as well as hTR are required to know which component might limit telomerase levels in a given cell type.

Here, we report fresh measurements of the number of hTR molecules in several immortalized human cells, including the telomerase-negative VA13 cell line, which maintains telomeres by the alternative lengthening of telomeres pathway (22). We also establish an approach to measure the number of hTERT protein molecules and assembled active telomerase RNPs per cell. Our results suggest hTR is in excess of hTERT, but each of these two subunits has a subpopulation or pool that is not assembled into active telomerase RNP. We hereby propose the model that telomerase assembly in human cells is an equilibrium process, with both hTR and hTERT as limiting factors. We also determine the specific activity and *K*_m_ for dGTP of telomerase and address a question that is much discussed in the telomerase field: whether ‘super-telomerase’, obtained by overexpression of hTR and hTERT in human cells, might have very different enzyme activity than endogenous telomerase. Our specific activity measurements suggest them to be equivalent, supporting the use of super-telomerase for biochemical studies.

## MATERIALS AND METHODS

### Cell culture

HEK 293T cells (ATCC), HeLa-EM2-11ht cells (Tet Systems Holdings GmbH & Co. KG) and VA13 cells (ATCC) were grown in high glucose Dulbecco's modified Eagle medium (DMEM) supplied with 10% fetal bovine serum (FBS), 2 mM GlutaMAX™-I (Life Technologies), 100 units/ml penicillin and 100 μg/ml streptomycin at 37°C with 5% CO_2_. Cells were harvested at ∼80% confluency for experiments.

### Preparation of standard hTR RNA

Sequence of hTR was inserted behind the T7 promoter of the pUC19 plasmid. The plasmid was linearized by cutting with FokI and then used as template for *in vitro* transcription with T7 RNA polymerase. The RNA products of the *in vitro* transcription were ethanol-precipitated and then gel-purified. Concentration of the standard RNA was determined with a NanoDrop spectrophotometer (Thermo).

### Preparation of standard hTERT protein

N-terminal 3×FLAG-tagged human TERT was expressed from phTERT-3×FLAG using the TNT^®^ Quick Coupled Transcription/Translation System (Promega) as previously described (23). Each reaction was performed with 400 μl TNT^®^ Quick Master Mix, 10 μl 1.0 mM l-methionine, 10 μl ^35^S-l-methionine (1 mCi in 98 μl, 1175 Ci/mmol, PerkinElmer), 10 μl T7 TNT^®^ PCR Enhancer, 10 μg phTERT-3×FLAG plasmid, 10 μg *in vitro* transcribed hTR (as described above) and nuclease-free water in a total volume of 500 μl. In the experiment of Supplementary Figure S3a, each reaction was performed in 100 μl and amounts of methionine used were as indicated in the figure. After incubation at 30°C for 1.5 h, 10 μl was removed as the input sample. The rest of the mixture was incubated with ANTI-FLAG^®^ M2 Affinity Gel (Sigma) at 4°C for 2 h to immunoprecipitate the reconstituted telomerase. The beads were then washed with 1× telomerase buffer A (50 mM Tris-HCl pH 8.0, 50 mM KCl, 1 mM MgCl_2_, 1 mM spermidine, 5 mM β-mercaptoethanol, 30% glycerol) four times, and then resuspended in the same buffer. ^35^S levels in the input and immunoprecipitated material were measured by liquid scintillation counting, and the amount of hTERT protein on the beads was calculated as described in Supplementary Materials. The radiolabeled hTERT protein was examined with sodium dodecylsulphate-polyacrylamide gel electrophoresis (SDS-PAGE). The signals were detected with a Typhoon Trio PhosphorImager (GE Healthcare) and quantified with ImageQuant TL v2005 software. The immunoprecipitated material was snap-frozen in liquid nitrogen and stored at −80°C.

### RNA extraction

Total RNA from different cells lines was extracted with TRIzol^®^ Reagent (Ambion) according to the manufacturer's instructions. RNA in hTERT immunoprecipitation elutions was extracted with TRIzol^®^ LS Reagent (Ambion) according to the manufacturer's instructions. Because the RNA level is low in the elution, yeast tRNA (Sigma, R563667, final concentration: 20 ng/μl) and glycogen (Roche, 10901393001, final concentration: 40 ng/μl) were added to help precipitation.

### RT-qPCR

RNA samples were treated with RQ1 RNase-free DNase (Promega) according to the manufacturer's instructions to eliminate genomic DNA contamination. cDNA was then prepared using the High Capacity cDNA Reverse Transcription kit (Applied Biosystems). RT-qPCR was performed with iQ™ SYBR^®^ Green Supermix (Bio-Rad) on the LightCycler^®^ 480 Real-Time PCR System (Roche). Sequences of the primers are listed in Supplementary Table S1. Polymerase chain reaction (PCR) products of the primers were examined with electrophoresis on a 3% agarose gel.

### Northern blot

RNA samples were mixed with equal volume of 2× formamide loading buffer (93% formamide, 0.1× Tris/Borate/EDTA (TBE), 30 mM EDTA, 0.03% bromophenol blue, 0.03% xylene cyanol), heated at 95°C for 5 min and then electrophoresed on a 4% polyacrylamide/7 M urea/1× TBE denaturing gel. Then the RNA was transferred onto a Hybond^TM^-N^+^ membrane (GE Healthcare) in 1× TBE at 1 A for 1–2 h, and cross-linked to the membrane under UV 254 nm at 1200 × 100 μJ/cm^2^. The membrane was pre-hybridized in Church buffer (0.5 M Na_2_HPO_4_-H_3_PO_4_ buffer pH 7.2, 1 mM EDTA, 7% SDS, 1% BSA) at 35°C for 30 min, then hybridized in Church buffer with 5′-end-labeled oligo probes (Supplementary Table S2) at 35°C overnight. After that, the membrane was washed once with 2× SSC, 0.1% SDS at 50°C for 20 min, then twice with 0.1× SSC, 0.1% SDS at 50°C for 20 min each time. The signals on the membrane were detected with a Typhoon Trio PhosphorImager (GE Healthcare) and quantified with ImageQuant TL v2005 software.

### Western blot

Protein samples were mixed with one-third volume of NuPAGE^®^ LDS Sample Buffer (4×) (Life Technologies), boiled at 95°C for 5 min, and then electrophoresed on a 4–12% Bis-Tris gel (Life Technologies). Standard SDS-PAGE and western blotting protocols were carried out afterwards. Primary antibodies used were as follows: anti-hTERT antibody (Abcam, ab32020, 1:1000), anti-β-actin antibody (Sigma, A5441, 1:5000). Secondary antibodies used were as follows: peroxidase-AffiniPure donkey anti-rabbit IgG (H + L) (Jackson, 711-035-152, 1:5000), peroxidase-AffiniPure donkey anti-mouse IgG (H + L) (Jackson, 715-035-150, 1:5000). SuperSignal^®^ West Pico Chemiluminescent Substrate (Thermo Scientific) was used to generate signals on western blots. The signals were detected with a FluorChem HD2 imaging system (Alpha Innotech) and quantified with ImageQuant TL v2005 software.

### Cell synchronization and flow cytometry

Double thymidine block: HeLa cells were first arrested by treatment with 2 mM thymidine (Sigma) for 18 h. Then the cells were released for 9 h and arrested again with 2 mM thymidine for 17 h. After release from the second thymidine block, cells were harvested every 3 h for a total 24-h time course. Flow cytometry: trypsinized cells were spun down at 200 × *g* for 5 min and washed once with phosphate buffered saline (PBS). Then the cells were fixed with ice-cold 70% ethanol, washed once with PBS containing 0.1% Triton X-100, and stained at 37°C for 1 h with modified Vindelov solution (5 μg/ml propidium iodide, 33 μg/ml RNase A in PBS containing 0.1% Triton X-100) as described in (24). Flow cytometry was performed on a BD Accuri™ C6 Flow Cytometer and the results were analyzed with Weasel software.

### Overexpression of super-telomerase or telomerase subunits in HEK 293T and HeLa cells

Plasmids expressing hTR and hTERT, which were a gift from J. Lingner (EPFL, Lausanne) (25), were transiently transfected into HEK 293T or HeLa cells with Lipofectamine^®^ 2000 Reagent (Invitrogen) as described in (25).

### Telomerase immunopurification and telomerase activity assay

Immunopurification and specific activity assays of overexpressed telomerase/endogenous telomerase were performed as described in (26,27) with some modifications. Generally, HEK 293T cells with or without transfection of hTR and hTERT expression vectors were harvested by trypsinization. If direct assay needs to be performed with cell lysates, the cells are lysed with ice-cold CHAPS lysis buffer (10 mM Tris-HCl pH 7.5, 1 mM MgCl_2_, 1 mM EGTA, 0.5% 3-[(3-cholamidopropyl)dimethylammonio]-1-propanesulfonate (CHAPS), 10% glycerol, 1 mM phenylmethylsulfonyl fluoride (PMSF), 1 mM dithiothreitol (DTT)) at 4°C for 1 h and then supplied with 300 mM KCl after lysis. In the experiment shown in Figure 5, cells were lysed with ice-cold telomerase buffer B (50 mM HEPES–KOH pH 8.0, 300 mM KCl, 2 mM MgCl_2_, 0.1% Triton X-100, 10% glycerol, 1 mM PMSF, 1 mM DTT) at 4°C for 30 min. Telomerase was immunoprecipitated with a sheep polyclonal anti-hTERT antibody, which was a gift from S. Cohen (University of Sydney, Sydney), and Protein G agarose beads (Roche). Then the peptide antigen of the antibody, which was also a gift from S. Cohen, was used to elute telomerase from the beads. Both CHAPS buffer with 300 mM KCl and telomerase buffer B listed above can be used as elution buffer. The specific activity measurement in Figure 5 is performed with telomerase buffer B as elution buffer. Telomerase activity assay was performed by adding 30 μl telomerase sample (cell lysate or IP elution), to a 20 μl assay buffer containing: 125 mM Tris-HCl pH 8.5, 2.5 mM MgCl_2_, 12.5 mM DTT, 2.5 mM spermidine, 1.5 mM EDTA, 2.5 mM dTTP, 2.5 mM dATP, 25 μM dGTP, 3 μl [α-^32^P]-dGTP (10 μCi/μl, 3000 Ci/mmol, Perkin-Elmer) and 2.5 μM a5 primer (5′-TTAGGGTTAGGGTTAGCGTTA-3′) (28). The reaction was carried out for 2 h at 37°C (unless stated otherwise), and then quenched by adding 250 μl 3.6 M NH_4_OAc with a 5′-^32^P-labeled a5 primer as loading control. The radioactivity of the loading control was determined by liquid scintillation counting. The products of the assay were phenol-extracted, ethanol-precipitated and then examined on a 10% polyacrylamide/7 M urea/1× TBE denaturing gel. The signals were detected with a Typhoon Trio PhosphorImager (GE Healthcare) and quantified with ImageQuant TL v2005 software.

**Figure 1.**
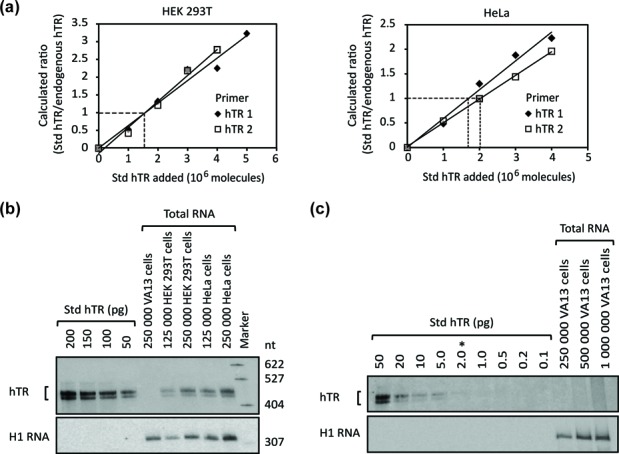
Quantification of endogenous hTR levels in HEK 293T, HeLa and VA13 cells. (**a**) Quantification of hTR levels by RT-qPCR with two primer pairs (hTR 1 and hTR 2). Different amounts of Std hTR were mixed with total RNA extracted from a known number of cells (∼2180 HEK 293T cells in the experiment using hTR 1, ∼2140 HEK 293T cells in experiment using hTR 2, ∼1470 HeLa cells in the experiments using hTR 1 and hTR 2). cDNA was prepared from each of these mixtures, and the total hTR level, consisting of both Std hTR and endogenous hTR, was measured with RT-qPCR, with GAPDH mRNA as internal control. The calculated ratio of Std hTR to endogenous hTR in each sample was plotted against the amount of Std hTR titrated in to make a standard curve. Dashed lines: the point on the standard curve at which the ‘calculated ratio’ axis value equals 1 corresponds to the amount of endogenous hTR in the sample on the ‘Std hTR added’ axis. (**b**) Quantification of hTR levels in HEK 293T, HeLa and VA13 cells by northern blot. The number of endogenous hTR molecules per cell for each cell line was obtained by comparing the northern blot signals from known numbers of Std hTR molecules with that from endogenous hTR present in the total RNA extracted from a known number of cells. H1 RNA (the RNA component of RNase P) was also probed as an internal control. Marker: radiolabeled pBR322/MspI (New England Biolabs), boiled in formamide loading buffer at 95°C for 5 min to be single-stranded. (**c**) Determination of the maximum possible hTR level in VA13 cells. Analysis was performed as in (b). *Detection limit of hTR by northern blot.

**Figure 2.**
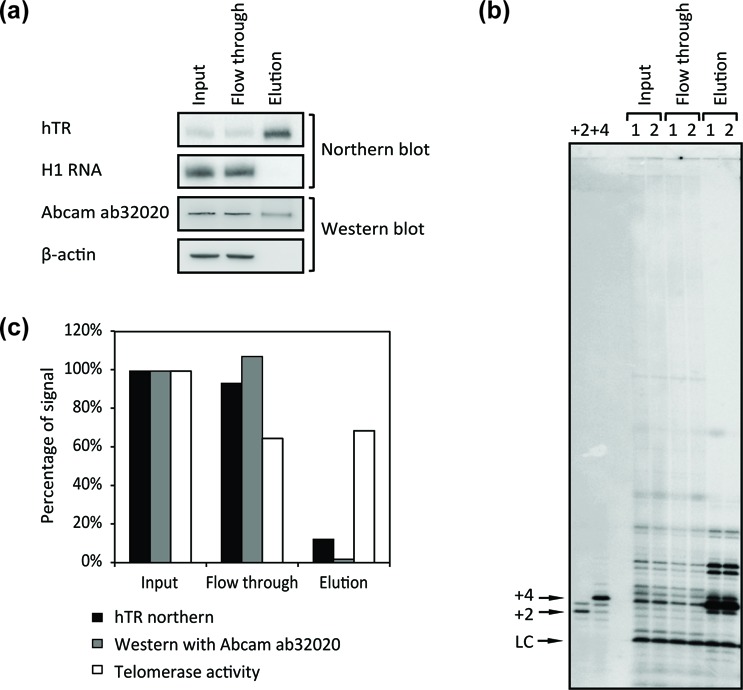
Analysis of hTR, hTERT and telomerase activity in the hTERT IP samples. (**a**) hTERT was immunoprecipitated from HEK 293T cell lysate and eluted from beads with excess amounts of the corresponding peptide antigen as described in ‘Materials and Methods’ section. hTR in the input (0.67%), flow through (0.67%) and elution (40%) fractions was examined by northern blot, with H1 RNA serving as an internal control. Western blot with Abcam ab32020 was performed on the input (0.1%), flow through (0.1%) and elution (6%) fractions, with β-actin serving as an internal control. (**b**) Telomerase activity in the input (1%), flow through (1%) and elution (7.5%) fractions was examined by the direct enzyme assay as described in ‘Materials and Methods’ section. (1, 2) Duplicate repeats of experiment. +2 and +4, size markers made by extending the DNA primer by two or four nucleotides. LC, labeled unextended primer, serving as a loading control. (**c**) Summary of hTR levels, Abcam ab32020 western signals and telomerase activity levels present in flow through and elution fractions, as normalized to input levels, in the specific experiment shown in (a) and (b). Note that the telomerase activity in the elution (∼69% input) is higher than that lost from input to flow through (∼35% input), probably because cellular factors present in input/flow through but not elution are inhibiting telomerase activity. Replicates of this experiment in HEK 293T and HeLa cells are summarized in Table 2.

**Figure 3.**
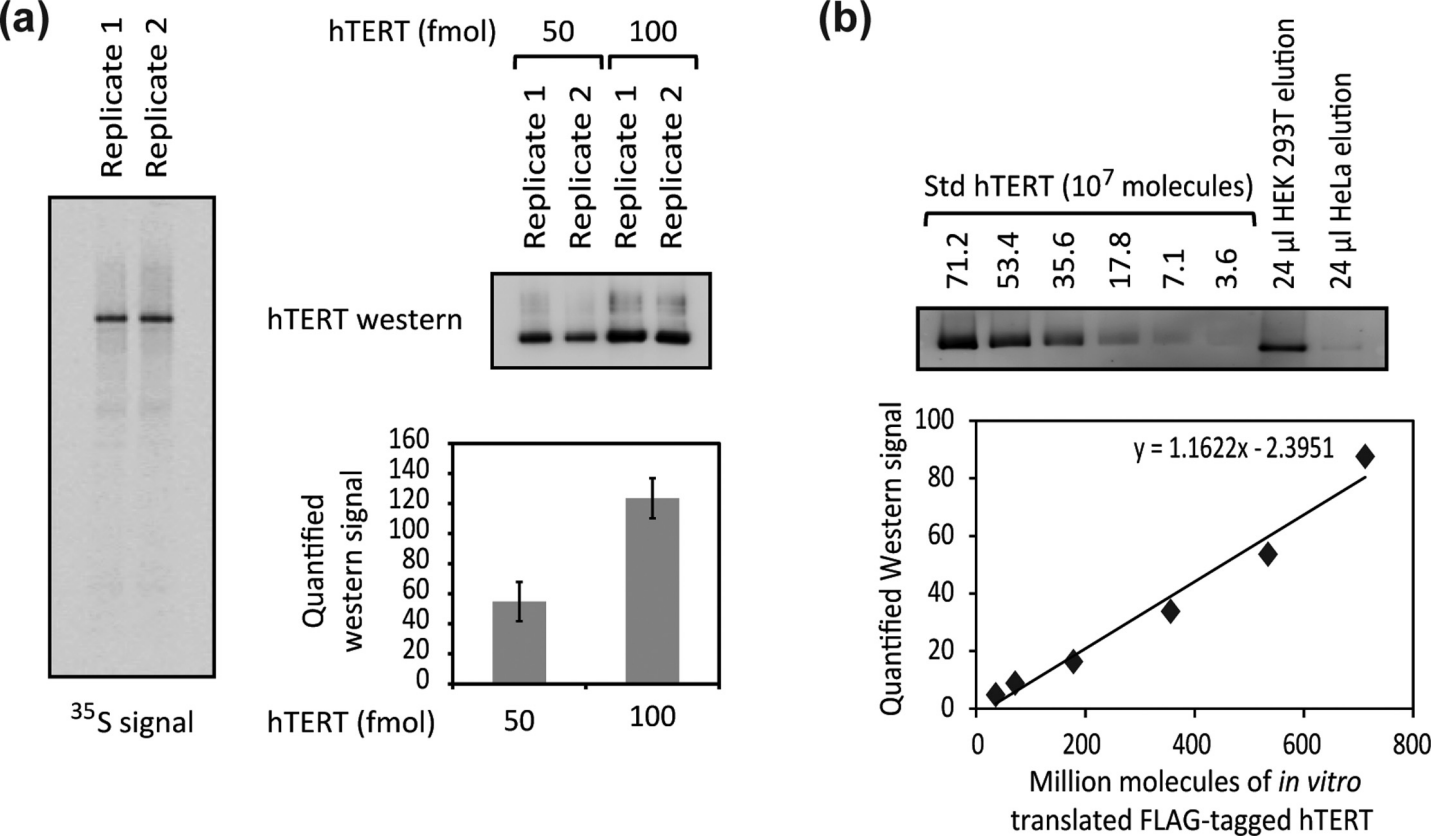
Quantification of hTERT protein levels in the hTERT IP elution samples of HEK 293T and HeLa cells. (**a**) Validation of the *in vitro* transcribed and translated hTERT protein for use as Std hTERT. Std hTERT from two independent preparations visualized by both ^35^S signal (left) and western blot (right). Quantification of the distribution of ^35^S signal in each lane (left) indicates that >90% of the radioactivity is in the band of FLAG-hTERT. Quantification of western blot signals is shown (error bars: SD, *n* = 2). (**b**) Quantification of hTERT protein levels in the hTERT IP elution samples of HEK 293T and HeLa cells by western blot. The number of hTERT molecules in the elution samples was calculated by comparing the western blot signals obtained from these samples to that from a known number of Std hTERT molecules. Note that we used a lower number of HeLa cells to prepare the input sample so the band in the HeLa IP elution is lighter that in the HEK 293T IP elution.

**Figure 4.**
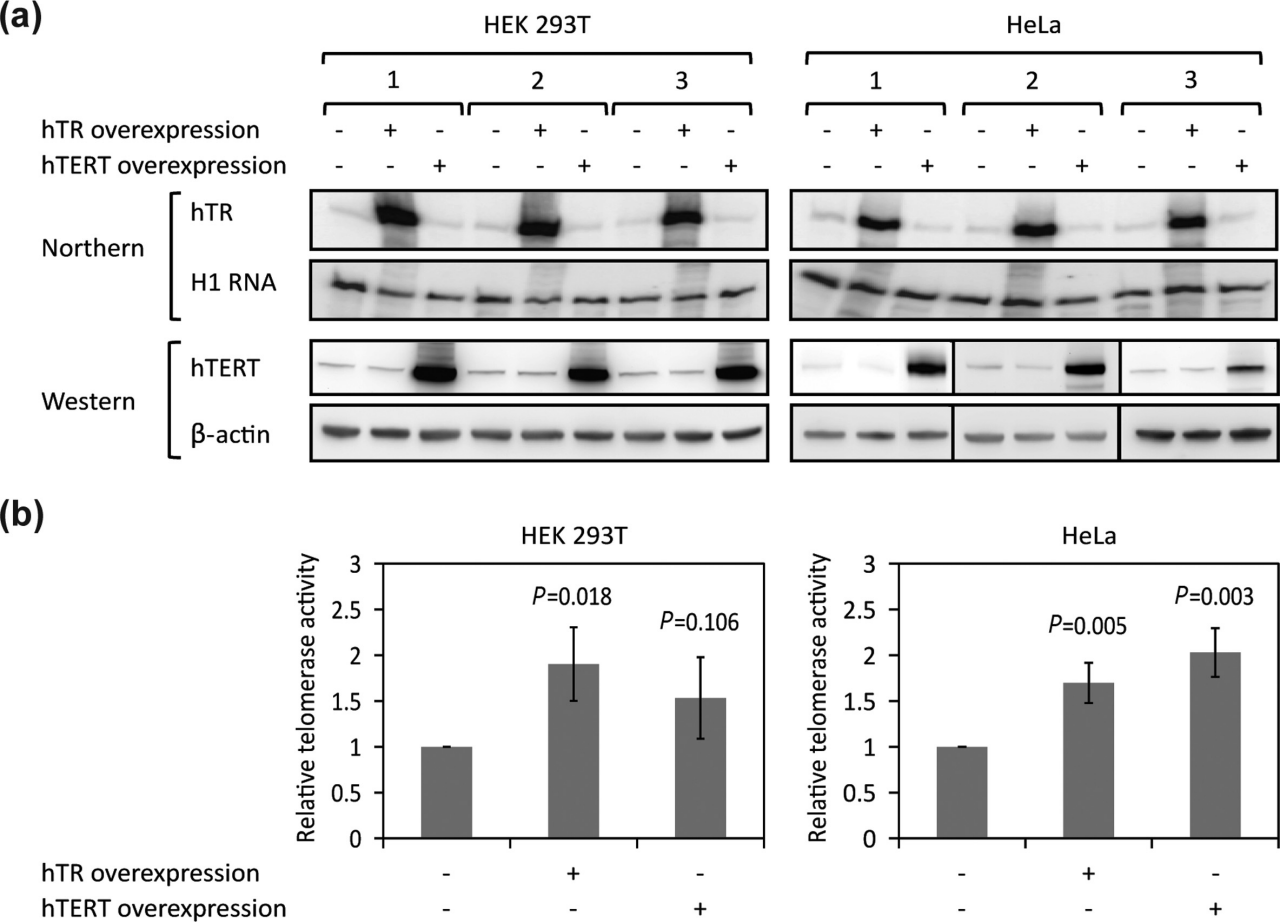
Overexpression of either hTR or hTERT increases total telomerase activity in living cells. (**a**) hTR or hTERT overexpression in HEK 293T and HeLa cells was quantified by northern or western blot, respectively, with H1 RNA or β-actin as loading controls. (**b**) Quantification of telomerase activity as measured by direct telomerase assay using lysates from HEK 293T or HeLa cells untransfected or transfected with either hTR or hTERT expression vectors (error bars: SD, *n* = 3).

**Figure 5.**
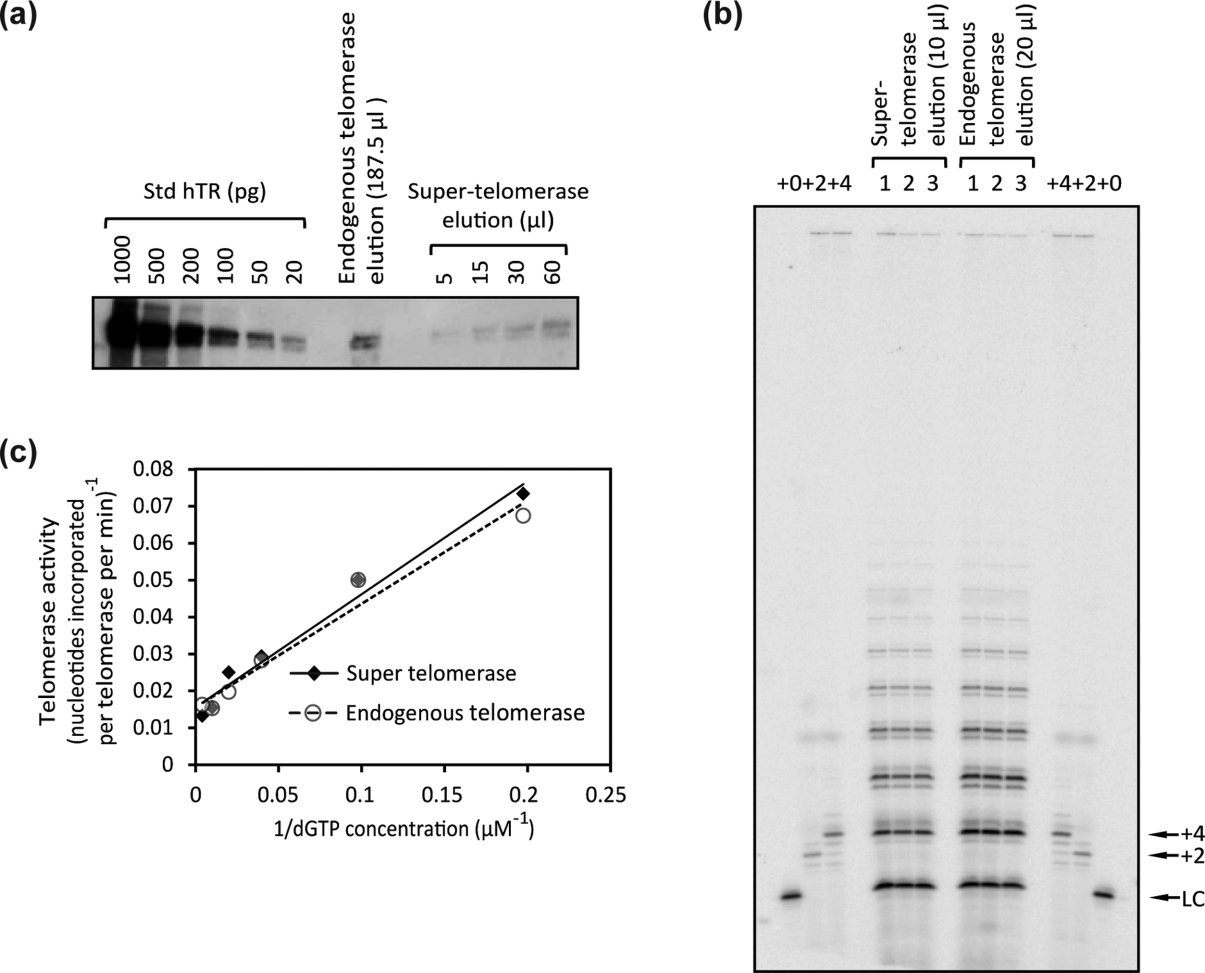
Measurement of the specific activity of endogenous telomerase and of super-telomerase. (**a**) Quantification of the hTR molecules co-immunoprecipitated with hTERT. The number of endogenous or overexpressed hTR molecules in the corresponding elution samples was measured by comparing northern blot signals obtained from a titration of Std hTR to that from hTR present in the indicated volumes of elutions. (**b**) Quantification of activity of telomerase eluted from antibody beads by the direct assay (see ‘Materials and Methods’ section). (1, 2, 3) Triplicate repeats of experiment. +0, labeled unextended primer, which also served as a loading control (LC). +2 and +4, size markers made by extending the DNA primer by two or four nucleotides. Signals of extension products on the gel were compared to that of the 18-mer LC to calculate the absolute radioactivity of the extension products. Given the number of hTR molecules in the elution samples measured in (a), the specific activity (nt incorporated per telomerase per minute) was then calculated. (**c**) Telomerase activity as a function of dGTP concentration shown as a Lineweaver–Burk plot. Each point is the average of two technical replicates. One of two biological replicates is shown. For super-telomerase, *V*_max_ = 57 ± 10 nt incorporated per telomerase monomer per minute, *K*_m_ = 17 ± 3 μM; for endogenous telomerase, *V*_max_ = 59 ± 8 nt incorporated per telomerase monomer per minute, *K*_m_ = 16 ± 2 μM (mean ± SD, *n* = 2 biological replicates.)

## RESULTS

### Quantification of endogenous hTR levels

We employed both reverse transcription-quantitative PCR (RT-qPCR) and quantitative northern blot analyses to assess the levels of endogenous hTR in HEK 293T, HeLa and VA13 cell lines. For RT-qPCR, four primer pairs targeting different regions of hTR were designed (Supplementary Table S1). The efficiency and specificity of each primer pair was validated (Supplementary Figure S1a and b). However, RT-qPCR on total RNA extracted from each cell line, with *in vitro* transcribed standard hTR (Std hTR) examined in separate reactions in parallel, gave hTR levels that differed by as much as 10-fold depending on which primer pair was used (data not shown). One possibility for such variation could be non-specific primer annealing to other cDNAs prepared from cellular RNA. Such non-specific annealing would not lead to extra PCR products, if the positions bound by the primers were not close enough, but would affect amplification efficiencies of the primers. To circumvent this concern, we titrated various amounts of Std hTR into total cellular RNA, and then prepared cDNA for RT-qPCR analysis from these mixtures, allowing the Std hTR and endogenous hTR to be reverse-transcribed and amplified in the same environment. The number of molecules of endogenous hTR was then calculated as described in Supplementary Materials. Employing this approach, we measured hTR abundances of 650–1260 molecules per HEK 293T cell and 1040–1840 molecules per HeLa cell; hTR was undetectable in the telomerase-negative VA13 cells. Sample data are shown in Figure 1a, and copy numbers measured by all four primer pairs are listed in Supplementary Table S3. The results obtained from different primer pairs were now very close to each other, with less than 2-fold differences observed amongst estimates.

To measure hTR copy number per cell with an independent method, we performed quantitative northern blot analysis (Figure 1b). The doublet of bands is characteristic of hTR (29), and may result from incomplete denaturation. Different amounts of Std hTR and total cellular RNA were loaded on the gel to confirm that the signals were within the linear range. By comparing the signals of Std hTR and endogenous hTR from a known number of cells, we estimate there are 750 ± 60 molecules of hTR per HEK 293T cell (mean ± SD, *n* = 3) and 1150 ± 150 molecules of hTR per HeLa cell (mean ± SD, *n* = 3). VA13, however, did not have a detectable level of hTR. To determine the detection limit of hTR by northern blot, and thus the upper limit of hTR molecules possibly present in VA13 cells, we titrated down the amount of Std hTR loaded onto the gel. As shown in Figure 1c, the detection limit of hTR by northern blot was 2 pg (equivalent to 8.3 × 10^6^ molecules). Because no signal was seen in total RNA from 1 million VA13 cells, there should be less than eight copies of hTR per VA13 cell. All hTR values measured for different cell lines are summarized in Table 1.

**Table 1.** Endogenous hTR levels in different cell lines

Cell line	hTR/cell (RT-qPCR)^a^	hTR/cell (northern blot)
HEK 293T	880 ± 290	750 ± 60^b^
HeLa	1440 ± 340	1150 ± 150^b^
VA13	Undetectable	Undetectable (<8)

^a^The hTR/cell (RT-qPCR) values of HEK 293T and HeLa are the average (±SD) of the values determined by the four RT-qPCR primer pairs as listed in Supplementary Table S1.

^b^Mean ± SD, *n* = 3.

### A two-step IP-western method for detecting endogenous hTERT

Previously, the hTERT monoclonal antibody Abcam ab32020, which recognizes a ∼130 kDa protein by western blot, was shown to recognize overexpressed hTERT in cell extracts with high efficiency and specificity, such that a single amino-acid substitution in the C-terminal epitope of hTERT abolished the signal (23). However, endogenous hTERT is not abundant, so a cross-reacting protein that does not interfere with western blots of overexpressed protein could become problematic for measuring the endogenous protein. We therefore employed the hTERT immunoprecipitation (IP) protocol established by Cohen and Reddel (26,27), in which endogenous hTERT was immunoprecipitated from cell lysate using a sheep polyclonal antibody recognizing a different epitope than Abcam ab32020, and then eluted with the corresponding peptide antigen. Western blot was performed on the input, flow through and elution samples with Abcam ab32020. To measure telomerase activities in these samples, instead of the PCR-based telomeric repeat amplification protocol (TRAP) assay, we employed the more accurate direct assay (23,30). In the direct assay, a telomeric DNA oligonucleotide substrate is extended by telomerase in the presence of [α-^32^P]-dGTP, and the extension products are visualized and quantified upon electrophoresis. By replacing telomerase buffer B in the original protocol with CHAPS lysis buffer (see ‘Materials and Methods’ section), robust detection and quantification of endogenous telomerase activity in cell lysate samples (input and flow through) was achieved.

Surprisingly, although a substantial amount of telomerase activity was depleted from the cell lysate after IP, the western signal of the ∼130 kDa protein detected by Abcam ab32020 was mostly unchanged (Figure 2 and Supplementary Figure S2). This led to the concern that another ∼130 kDa protein in the cell lysate cross-reacts with Abcam ab32020, in which case Abcam ab32020 is not useful for identifying endogenous hTERT in cell lysates. (Other antibodies were tested and found to have even lower specificity for hTERT on western blots; A. J. Zaug and T.R.C., unpublished.) However, we reasoned that the ∼130 kDa protein band in the IP elution detected by Abcam ab32020 should specifically reveal endogenous hTERT because any contaminating, cross-reacting protein should not be immunoprecipitated with the IP antibody, which recognizes a completely different epitope, nor be eluted by the peptide antigen corresponding to the epitope of the IP antibody. Consistent with this expectation, the ∼130 kDa band recognized by Abcam ab32020 became highly enriched compared to another endogenous protein, β-actin, in the elution volume as compared to in bulk lysate (Figure 2a). Thus, this two-step IP-western protocol allows specific detection of endogenous hTERT.

### Quantification of endogenous levels of hTERT and telomerase RNP

Examination of hTR in the input and flow through samples of the hTERT IP with northern blot revealed that the percentage of hTR depleted from the cell lysate by IP is less than that of the telomerase activity depleted (Figure 2c and Table 2), which suggests only a portion of the hTR population is assembled into active telomerase RNP. Comparing the percentage of hTR depleted to that of telomerase activity depleted, we calculated the percentage of hTR that is assembled into telomerase RNP. Combined with the number of hTR molecules per cell measured before, the number of telomerase RNP monomers per cell can be quantified. With this analysis, we estimate ∼240 active telomerase monomers per cell for both HEK 293T and HeLa cells (Table 2). If telomerase is a dimer (27,31), then there would be half this number of telomerase dimers per cell. Note that this calculation (see Table 2 legend) does not depend on the absolute efficiency of the IP.

**Table 2.** Endogenous assembled telomerase and hTERT protein levels in different cell lines

Cell line	HEK 293T			HeLa		
Replicate number	1	2	3	1	2	3
Telomerase activity ratio (flow through/input)	0.65	0.48	0.44	0.56	0.37	0.33
hTR ratio (flow through/input)	0.94	0.84	0.77	0.91	0.88	0.88
Estimated number of assembled telomerase monomer per cell^a^	146	245	332	255	255	222
Million hTR per μl elution^b^	9.2	8.2		1.2	1.3	1.4
Million hTERT per μl elution	15	11		1.7	3.1	3.3
Estimated number of hTERT protein per cell^c^	237	322		382	608	534

^a^The number of telomerase monomers per cell is calculated as:

number of hTR RNA per cell × [1 − hTR ratio (flow through/input)]/[1 − telomerase activity ratio (flow through/input)]

We use 800 copies of hTR RNA per HEK 293T cell and 1300 copies of hTR RNA per HeLa cell for the calculation. Based on the numbers in the table, HEK 293T has 240 ± 90 telomerase monomers per cell (mean ± SD, *n* = 3); HeLa has 240 ± 20 telomerase monomers per cell (mean ± SD, *n* = 3).

^b^hTR levels in the elution are measured by RT-qPCR with the primer pair hTR 1.

^c^The number of hTERT protein per cell is calculated as:

Number of telomerase monomer per cell × number of hTERT per μl elution/number of hTR per μl elution

Based on the numbers in the table, HEK 293T has 280 ± 60 hTERT molecules per cell (mean ± SD, *n* = 2); HeLa has 500 ± 110 hTERT molecules per cell (mean ± SD, *n* = 3).

We then quantified the endogenous hTERT levels taking advantage of the quantified telomerase RNP levels. Because hTR would only be immunoprecipitated with the IP antibody and eluted with the corresponding peptide antigen when assembled with hTERT, the number of hTR molecules should equal the number of telomerase monomers in the IP elution. Assuming an equal affinity of the IP antibody for all forms of endogenous hTERT (hTERT in telomerase RNP + hTERT not bound to hTR) in the cell lysate, the molecular ratio of hTR to hTERT in the elution should reflect the ratio of telomerase RNP to total hTERT in the cell, allowing the number of hTERT molecules per cell to be calculated.

To quantify the number of hTERT protein molecules in the elution, an hTERT protein standard (Std hTERT) is required. Std hTERT was prepared by reconstituting telomerase in rabbit reticulocyte lysates in the presence of ^35^S-methionine and using the ^35^S signal to calculate the number of hTERT molecules (Supplementary Materials). We addressed two potential concerns by showing (i) almost all of the radioactivity in the immunoprecipitated material is in the protein band, so the background does not appreciably affect the quantification of Std hTERT protein (Figure 3a and Supplementary Figure S3a) and (ii) the pre-existing methionine concentration in the RRLs is negligible compared to the exogenously supplemented concentration, and thus would not significantly affect the estimation of protein yields (Supplementary Figure S3a). Quantitative western blot analysis was then performed on hTERT IP elution samples from HEK 293T and HeLa cell lysates, using Std hTERT to provide a standard curve (Figure 3b). hTR levels in the elution samples were quantified through RT-qPCR. Combined with the number of telomerase RNP per cell quantified above, we estimated 280 ± 60 hTERT protein molecules per HEK 293T cell (mean ± SD, *n* = 2) and 500 ± 110 hTERT protein molecules per HeLa cell (mean ± SD, *n* = 3) (Table 2). This result suggests that hTERT has a population that is not assembled into telomerase RNP in the case of HeLa cells (*P* = 0.027, one-tailed *t*-test). The uncertainty of the measurements does not allow a firm conclusion for HEK 293T cells, but the trend was in the same direction (hTERT > telomerase) in both experiments in Table 2.

It needs to be pointed out the quantification analysis above was performed on an asynchronous cell population, while telomerase activity has been shown to vary in different phases of the cell cycle (32), and cell cycle-dependent regulation of TR and TERT in different species has been reported (33,34). Hence, we examined the endogenous levels of hTR and hTERT mRNA levels as a function of cell cycle progression. HeLa cells were synchronized at early S phase with double thymidine block. Then a 24-h follow-up examination was performed after the cells were released from the block. Cell cycle progression was analyzed by flow cytometry (Supplementary Figure S4a) and levels of hTR and hTERT mRNA were measured by RT-qPCR using U6 snRNA or 18S rRNA as internal controls (Supplementary Figure S4b, c, verification of the hTERT RT-qPCR primers is shown in S1c and d). hTR level was seen to be fairly stable throughout the cell cycle. However, hTERT mRNA levels increased in late S/G2 phase and diminished during M/G1 phase, indicating hTERT protein levels might be dynamic during cell cycle progression.

### Overexpression of either hTR or hTERT alone increases total telomerase activity, suggesting that a subpopulation of each subunit is not assembled into telomerase RNP in cells

Quantification of endogenous hTR, hTERT and active telomerase RNP levels above indicates that the number of active telomerase RNPs per cell is less than the number of either hTR or hTERT subunits. Consistent with this result, overexpressing either subunit alone was previously shown to increase total telomerase activity in both HEK 293T and HeLa cells, as measured by the TRAP assay (25). However, as the TRAP assay can be unreliable (23,30), we examined how overexpression of either hTR or hTERT affected total telomerase activity within cells, employing the direct enzyme assay described above.

Northern and western blot, respectively, revealed a 40- to 90-fold increase in hTR levels and a >10-fold increase in hTERT levels upon overexpression in either HEK 293T or HeLa cells by transient transfection. The 10-fold increase in hTERT expression is a lower limit of estimation due to the probable presence of the other ∼130 kDa protein in cell lysates that cross-reacts with the Abcam ab32020 detection antibody (Figure 4a). The direct telomerase assay was performed on the cell lysates, and the results confirmed that overexpression of either subunit alone gave increased total telomerase activity (Figure 4b). On the basis of these experiments, it appears that both HEK 293T and HeLa cells contain subpopulations of unassembled hTR and hTERT that are approximately the same size as the pool of assembled hTR–hTERT RNP complexes (see ‘Discussion’ section below).

### Specific activity of endogenous telomerase and of overexpressed telomerase

As discussed above, the number of hTR molecules eluted after hTERT IP can serve as a proxy for the number of assembled telomerase monomers, and telomerase activity in the elution is measurable by the direct assay. Therefore we were able to determine the specific activity of the endogenous telomerase RNP as well as that of super-telomerase RNP, which was assembled in HEK 293T cells by overexpression of both hTR and hTERT subunits as previously reported (25). Lysates were prepared from HEK 293T cells with or without transfection of hTR and hTERT expression vectors, followed by the IP and elution steps described earlier. The concentrations of eluted endogenous and overexpressed hTR, and thus of eluted endogenous and super-telomerase monomer, were measured by quantitative northern blot to be ∼9 × 10^5^ molecules per microliter and ∼1.4 × 10^6^ molecules per microliter, respectively (Figure 5a). For these experiments, lysate prepared from cells expressing super-telomerase was diluted such that the final telomerase concentration was comparable to that in the lysate prepared from untransfected cells.

In order to measure the absolute number of nucleotides (nt) incorporated by a given amount of telomerase within a specific time span, an 18-mer loading control (LC), the absolute radioactivity of which was measured by liquid scintillation counting, was used as a standard to calibrate the extension products of the direct assay after electrophoresis. Comparison of the LC signal with that of the extension products allows for a calculation of how many molecules of [α-^32^P]-dGMP, and correspondingly how many total nucleotides, were incorporated during the reaction. We validated this approach by showing that (i) ∼90% of the radioactivity of the LC sample was in the position of the 18-mer band (Supplementary Figure S5a), a number which was taken into consideration for the calculation, (ii) the amount of extension products increased linearly from 0 to 2.5 h (Supplementary Figure S5b), indicating that there was no loss in enzymatic activity during the course of the experiment and (iii) the amount of extension products increased linearly as more purified telomerase was added, indicating that the concentrations of enzyme used in these experiments were not at saturating levels (Supplementary Figure S5c). Based on analysis of the data shown in Figure 5a and b, we estimate the specific activities of endogenous telomerase and of super-telomerase to be 22.4 ± 0.2 and 19.3 ± 0.1 nt incorporated per telomerase monomer per minute respectively, using the conditions described in ‘Materials and Methods’ section. Considering the great number of steps involved in obtaining these values, which could conceivably lead to systematic errors, we conclude that there is no substantial difference in the specific activities of endogenous and super-telomerase, and we estimate the specific activity of both enzymes under these specific conditions to be 20 ± 5 nt incorporated per enzyme active site per minute. Recognizing that the low dGTP concentration used in these conditions (10 μM) may have limited the rate of extension by the enzyme, we titrated increasing concentrations of dGTP for both endogenous telomerase and super telomerase reactions (Figure 5c). Kinetic analysis revealed that both enzymes have a *K*_m_ of ∼17 μM for dGTP, and that the maximum specific activity for each under the conditions of the assay is ∼60 nt incorporated per telomerase monomer per minute.

## DISCUSSION

Two conclusions of biological interest emerge from our inventory of telomerase components in human cells. First, ∼240 telomerase RNP monomers per cell is very low, similar to the number of telomeres in late S phase. Telomerase dimers (27,31) would be present at only half the level of telomeres. Given that telomerase is tethered to its telomeric DNA substrate by interactions with the TPP1 component of shelterin (1), each telomerase may have limited opportunity to extend multiple telomeres per cell cycle. Thus, this low measured ratio of telomerase to telomeres makes it easy to understand the haploinsufficiency seen in human diseases, where one defective or partially defective allele of hTR or hTERT leads to failure to maintain telomeres and premature replicative senescence (23,35–37). Second, there are substantial subpopulations or pools of ‘free’ hTR and hTERT in cells that are competent and available to form active telomerase enzyme upon overexpression of the other component. The word ‘free’ is used to mean free of the telomerase RNP; certainly these molecules could be present in other macromolecular complexes. Because hTERT has been shown to have non-telomerase functions (38), a pool of free hTERT would be expected. A pool of free hTR might function in allowing increased hTERT to quickly assemble into active telomerase in late S phase, when telomeres are replicated. Another conceivable subpopulation of hTERT or hTR might be molecules that are normally degraded, because they are not stabilized in an RNP, and then upon increased expression of the other component they form stable RNPs; in this case, there might not be much of a pool of free molecules in the steady state.

### Quantification of telomerase subunits and RNP abundance: results and experimental caveats

In this study, we established methods to quantify the endogenous levels of hTR, hTERT protein and the assembled telomerase RNP in immortalized human cell lines, about which uncertainty still exists in the field. Previously, substantially different estimations of hTR copy number per cell (∼60 000 versus ∼120) were reported (14,15). Quantification of hTERT, on the other hand, has been hampered by the difficulty to prepare standard molecules and to detect the lowly expressed endogenous protein. The number of assembled telomerase RNPs was estimated to be 20–50 monomers per HEK 293 cell (27), based on calculations that relied on roughly estimated recovery efficiencies for several purification steps. Here, we adapted the absolute RT-qPCR procedure to make more accurate measurements of hTR levels, which were further confirmed with independent measurements by northern blot. For hTERT quantification, we developed a method to prepare Std hTERT molecules with the RRL telomerase reconstitution system and to detect endogenous hTERT protein in the telomerase IP elution samples. Our examination of telomerase RNP abundance was based on direct measurements of hTR and telomerase activity levels in input and flow through samples of the telomerase IP, and our examination of hTERT abundance was based on direct measurements of hTR and hTERT levels in elution samples of the IP. Neither of these calculations requires an estimate of IP recovery efficiencies. These quantitative analyses further suggest the existence of subpopulations of both hTR and hTERT not assembled into telomerase RNPs. Overexpression of either subunit alone resulted in increased telomerase activity, presumably because there was already available a free pool of the corresponding binding partner for RNP assembly or because free subunits that would normally be degraded were now incorporated into stable RNPs.

It should be pointed out that certain technical limits could introduce inaccuracies in our quantification. For example, for hTR quantification, some RNA could have been lost during the extraction process, resulting in an underestimate of hTR levels. Secondly, for hTERT examination, quantification of western blots by chemiluminescence is admittedly not as sensitive or as precise as the radioisotopic-based methods used to quantify hTR levels and telomerase activity. Furthermore, during the course of these studies we were surprised to discover that the monoclonal anti-hTERT antibody used for western blot detection (Abcam ab32020), which shows such exquisite specificity for measuring overexpressed hTERT, appears to cross-react with another endogenous protein of molecular weight similar to hTERT. (An alternative explanation for the ∼130 kDa protein would be the existence of a pool of hTERT protein in the cell that is not assembled into active telomerase nor immunoprecipitated by the anti-hTERT IP antibody. In this case, both the endogenous level of hTERT and the pool of hTERT not assembled into the telomerase RNP would be about 9000 molecules/HEK 293T cell and 20 000 molecules/HeLa cell (Supplementary Figure S3b), much higher than our estimations in Table 2.)

Unlike the case in HeLa cells, the precision of our measurements of hTR and hTERT levels in the IP elution samples for HEK 293T cells was insufficient to conclude that the hTERT protein was in excess over telomerase RNP, although the trend was in that direction (Table 2). However, given reports of telomerase-independent functions of hTERT (38) and the observation that hTR overexpression alone leads to increased telomerase activity (Figure 4), it is likely that there does indeed exist a subpopulation of hTERT which is not assembled in telomerase in HEK 293T cells as well. Despite our concerns with the use of western blots for hTERT quantification, our estimate of hTERT abundance is reasonably close to the previous estimate of <600 copies of hTERT protein per H1299 cancer cell (14).

### Telomerase: a non-abundant RNP

In our study, we estimate the copy number of the telomerase monomer per HEK 293T and per HeLa cell to be ∼240, which suggests there are fewer telomerase active sites than chromosomal ends at late S phase in an HEK 293T cell (256 telomeres) or in a HeLa cell (304–320 telomeres) (16,39). Fewer telomerase molecules that telomeres is consistent with the work of Cao *et al.* (15), who estimated 20–50 telomerases per HEK 293 cell. Fewer telomerase active sites than telomeres is also the case with budding yeast (13). Taking into consideration that telomerase levels could fluctuate throughout the cell cycle (Supplementary Figure S4), with potentially the highest levels occurring in late S phase, it is possible that during DNA synthesis telomerase is upregulated to levels stoichiometric to the number of chromosome ends. Regardless, telomerase is certainly a non-abundant RNP when compared to other RNPs such as the small nuclear RNPs (snRNPs), 7SK RNP and RNase P (Table 3) (40). This is not surprising because these other RNPs function in processes of pre-mRNA transcription and splicing and tRNA and rRNA maturation; these processes involve many more substrates and occur with much greater frequency than the process of telomere extension. Telomerase abundance is still lower than, but much closer to, the levels of other small Cajal body-specific RNPs (scaRNPs) (Table 3).

**Table 3.** Comparison of endogenous human telomerase levels with the levels of other ribonucleoprotein complexes

RNP	Copies/cell^a^
Telomerase^b^	2 × 10^2^
U1	1 × 10^6^
U2	5 × 10^5^
U4	2 × 10^5^
U5	2 × 10^5^
U6	4 × 10^5^
U7	4 × 10^3^
Box C/D scaRNPs	1 × 10^3^
Box H/ACA scaRNPs	1 × 10^3^
Box C/D-H/ACA scaRNPs	1 × 10^3^
7SK	2 × 10^5^
RNase P	2 × 10^5^

^a^Copy numbers of the non-telomerase RNPs are from (40).

^b^The number shown here is that of telomerase monomer, which is the assembly of one hTR and one hTERT molecule. The copy number of telomerase dimers would be half of the number shown here.

### Equilibrium model for telomerase assembly

Overexpression of either hTR or hTERT alone, in HEK 293T or HeLa cells, resulted in increased telomerase activity (Figure 4), and our quantitative analysis also indicates that there are more hTR and (in HeLa) hTERT subunits per cell than there are assembled telomerase RNPs. A simple model to explain these observations is that the assembly of hTR and hTERT into active telomerase is an equilibrium process; then, according to L'Chatelier's principle, increasing the concentration of either subunit would drive the reaction towards increased RNP assembly. Such a model predicts that both hTR and hTERT are limiting factors for assembly, which would explain why individuals heterozygotic for either hTR or hTERT loss-of-function alleles are susceptible to diseases affecting highly proliferative tissues due to telomerase haploinsufficiency (23,35–37).

We note that the number of unassembled hTERT molecules in the HEK 293T/HeLa cells seems small based on our quantification. Taking the possible experimental caveats discussed above into consideration, we do not exclude the possibility that hTERT molecules stably present in these cells are actually all assembled into telomerase and that the subpopulation of hTERT that gets assembled upon hTR overexpression to increase total telomerase activity consists of molecules that are normally degraded, because they are not stabilized in an RNP. Besides, the quantitative analyses and overexpression experiments described here were both done in cell populations that could be heterogeneous with respect to the actual molecular ratio of these telomerase components; furthermore, transfection and expression efficiencies of hTR and hTERT expression vectors could be variable among cells. It is thus difficult to deduce the exact size of the telomerase-free hTR and hTERT populations within these cells.

### Relative specific activities of telomerase and super-telomerase

In addition to measuring the levels of telomerase components, we also determined the specific activity of both the endogenous and overexpressed enzymes. Our measurements demonstrate that endogenous telomerase and super-telomerase have equal specific activities and equal *K*_m_(dGTP) values. These results support the use of super-telomerase in experiments designed to study the endogenous enzyme. The maximum specific activity observed under the conditions described in ‘Materials and Methods’ section was ∼60 nt incorporated per enzyme per minute. This is of the same magnitude as that calculated by measuring the rate of extension of oligonucleotide substrates under processive extension conditions (23,41–43). A comparison of the enzymatic activity of telomerase to that of other nucleic acid polymerases (Table 4) (44–47) indicates that telomerase is not a very efficient polymerase. Furthermore, the cellular dGTP concentration is in the range of ∼1.5 μM (48), which is below *K*_m_ and therefore should render its activity even lower. However, such low activity is not unexpected considering that under conditions of telomere length maintenance, each telomere is extended by only ∼65 nt by a single enzyme during each cell division (49,50). In other words, there is no selective pressure to improve an inefficient enzyme if its catalytic capacity is sufficient to perform its function.

**Table 4.** Comparison of the specific activity of human telomerase with those of other nucleic acid polymerases

Enzyme	Specific activity (nucleotides/enzyme/min)	Reference
Telomerase	∼60	This work
HIV-1 reverse transcriptase	∼1100	(44)
Avian reverse transcriptase	1200–1800	(45)
Human RNA polymerase II	∼1200	(46)
*E. coli* DNA polymerase III	∼54 000	(47)

## SUPPLEMENTARY DATA

Supplementary DATA are available at NAR Online.

SUPPLEMENTARY DATA

## References

[1] Nandakumar J., Cech T.R. (2013). Finding the end: recruitment of telomerase to telomeres. Nat. Rev. Mol. Cell Biol..

[2] Jaskelioff M., Muller F.L., Paik J.H., Thomas E., Jiang S., Adams A.C., Sahin E., Kost-Alimova M., Protopopov A., Cadinanos J. (2011). Telomerase reactivation reverses tissue degeneration in aged telomerase-deficient mice. Nature.

[3] Armanios M., Blackburn E.H. (2012). The telomere syndromes. Nat. Rev. Genet..

[4] Meyerson M. (2000). Role of telomerase in normal and cancer cells. J. Clin. Oncol..

[5] Greider C.W., Blackburn E.H. (1989). A telomeric sequence in the RNA of Tetrahymena telomerase required for telomere repeat synthesis. Nature.

[6] Nakamura T.M., Morin G.B., Chapman K.B., Weinrich S.L., Andrews W.H., Lingner J., Harley C.B., Cech T.R. (1997). Telomerase catalytic subunit homologs from fission yeast and human. Science.

[7] Meyerson M., Counter C.M., Eaton E.N., Ellisen L.W., Steiner P., Caddle S.D., Ziaugra L., Beijersbergen R.L., Davidoff M.J., Liu Q. (1997). hEST2, the putative human telomerase catalytic subunit gene, is up-regulated in tumor cells and during immortalization. Cell.

[8] Mitchell J.R., Wood E., Collins K. (1999). A telomerase component is defective in the human disease dyskeratosis congenita. Nature.

[9] Egan E.D., Collins K. (2012). Biogenesis of telomerase ribonucleoproteins. RNA.

[10] Venteicher A.S., Abreu E.B., Meng Z., McCann K.E., Terns R.M., Veenstra T.D., Terns M.P., Artandi S.E. (2009). A human telomerase holoenzyme protein required for Cajal body localization and telomere synthesis. Science.

[11] Tycowski K.T., Shu M.D., Kukoyi A., Steitz J.A. (2009). A conserved WD40 protein binds the Cajal body localization signal of scaRNP particles. Mol. Cell.

[12] Takai K.K., Hooper S., Blackwood S., Gandhi R., de Lange T. (2010). In vivo stoichiometry of shelterin components. J. Biol. Chem..

[13] Mozdy A.D., Cech T.R. (2006). Low abundance of telomerase in yeast: implications for telomerase haploinsufficiency. RNA.

[14] Yi X., Shay J.W., Wright W.E. (2001). Quantitation of telomerase components and hTERT mRNA splicing patterns in immortal human cells. Nucleic Acids Res..

[15] Cao Y., Huschtscha L.I., Nouwens A.S., Pickett H.A., Neumann A.A., Chang A.C., Toouli C.D., Bryan T.M., Reddel R.R. (2008). Amplification of telomerase reverse transcriptase gene in human mammary epithelial cells with limiting telomerase RNA expression levels. Cancer Res..

[16] Bylund L., Kytola S., Lui W.O., Larsson C., Weber G. (2004). Analysis of the cytogenetic stability of the human embryonal kidney cell line 293 by cytogenetic and STR profiling approaches. Cytogenet. Genome Res..

[17] Feng J., Funk W.D., Wang S.S., Weinrich S.L., Avilion A.A., Chiu C.P., Adams R.R., Chang E., Allsopp R.C., Yu J. (1995). The RNA component of human telomerase. Science.

[18] Bodnar A.G., Ouellette M., Frolkis M., Holt S.E., Chiu C.P., Morin G.B., Harley C.B., Shay J.W., Lichtsteiner S., Wright W.E. (1998). Extension of life-span by introduction of telomerase into normal human cells. Science.

[19] Avilion A.A., Piatyszek M.A., Gupta J., Shay J.W., Bacchetti S., Greider C.W. (1996). Human telomerase RNA and telomerase activity in immortal cell lines and tumor tissues. Cancer Res..

[20] Weng N., Levine B.L., June C.H., Hodes R.J. (1997). Regulation of telomerase RNA template expression in human T lymphocyte development and activation. J. Immunol..

[21] Liu K., Hodes R.J., Weng N. (2001). Cutting edge: telomerase activation in human T lymphocytes does not require increase in telomerase reverse transcriptase (hTERT) protein but is associated with hTERT phosphorylation and nuclear translocation. J. Immunol..

[22] Bryan T.M., Marusic L., Bacchetti S., Namba M., Reddel R.R. (1997). The telomere lengthening mechanism in telomerase-negative immortal human cells does not involve the telomerase RNA subunit. Hum. Mol. Genet..

[23] Zaug A.J., Crary S.M., Jesse Fioravanti M., Campbell K., Cech T.R. (2013). Many disease-associated variants of hTERT retain high telomerase enzymatic activity. Nucleic Acids Res.

[24] Andrysik Z., Kim J., Tan A.C., Espinosa J.M. (2013). A genetic screen identifies TCF3/E2A and TRIAP1 as pathway-specific regulators of the cellular response to p53 activation. Cell Rep..

[25] Cristofari G., Lingner J. (2006). Telomere length homeostasis requires that telomerase levels are limiting. EMBO J..

[26] Cohen S.B., Reddel R.R. (2008). A sensitive direct human telomerase activity assay. Nat. Methods.

[27] Cohen S.B., Graham M.E., Lovrecz G.O., Bache N., Robinson P.J., Reddel R.R. (2007). Protein composition of catalytically active human telomerase from immortal cells. Science.

[28] Wang F., Podell E.R., Zaug A.J., Yang Y., Baciu P., Cech T.R., Lei M. (2007). The POT1-TPP1 telomere complex is a telomerase processivity factor. Nature.

[29] Mitchell J.R., Cheng J., Collins K. (1999). A box H/ACA small nucleolar RNA-like domain at the human telomerase RNA 3′ end. Mol. Cell. Biol..

[30] Podlevsky J.D., Chen J.J. (2012). It all comes together at the ends: telomerase structure, function, and biogenesis. Mutat. Res..

[31] Sauerwald A., Sandin S., Cristofari G., Scheres S.H., Lingner J., Rhodes D. (2013). Structure of active dimeric human telomerase. Nat. Struct. Mol. Biol..

[32] Zhu X., Kumar R., Mandal M., Sharma N., Sharma H.W., Dhingra U., Sokoloski J.A., Hsiao R., Narayanan R. (1996). Cell cycle-dependent modulation of telomerase activity in tumor cells. Proc. Natl. Acad. Sci. U.S.A..

[33] Jung H.Y., Wang X., Jun S., Park J.I. (2013). Dyrk2-associated EDD-DDB1-VprBP E3 ligase inhibits telomerase by TERT degradation. J. Biol. Chem..

[34] Dionne I., Larose S., Dandjinou A.T., Abou Elela S., Wellinger R.J. (2013). Cell cycle-dependent transcription factors control the expression of yeast telomerase RNA. RNA.

[35] Armanios M., Chen J.L., Chang Y.P., Brodsky R.A., Hawkins A., Griffin C.A., Eshleman J.R., Cohen A.R., Chakravarti A., Hamosh A. (2005). Haploinsufficiency of telomerase reverse transcriptase leads to anticipation in autosomal dominant dyskeratosis congenita. Proc. Natl. Acad. Sci. U.S.A..

[36] Trudeau M.A., Wong J.M. (2010). Genetic variations in telomere maintenance, with implications on tissue renewal capacity and chronic disease pathologies. Curr. Pharmacogenomics Pers. Med..

[37] Yamaguchi H., Calado R.T., Ly H., Kajigaya S., Baerlocher G.M., Chanock S.J., Lansdorp P.M., Young N.S. (2005). Mutations in TERT, the gene for telomerase reverse transcriptase, in aplastic anemia. N. Engl. J. Med..

[38] Chiodi I., Mondello C. (2012). Telomere-independent functions of telomerase in nuclei, cytoplasm, and mitochondria. Front. Oncol..

[39] Macville M., Schrock E., Padilla-Nash H., Keck C., Ghadimi B.M., Zimonjic D., Popescu N., Ried T. (1999). Comprehensive and definitive molecular cytogenetic characterization of HeLa cells by spectral karyotyping. Cancer Res..

[40] Tycowski K.T., Kolev N.G., Conrad N.K., Fok V., Steitz J.A., Gesteland R.F., Cech T.R., Atkins J.F. (2006). The ever-growing world of small nuclear ribonucleoproteins. The RNA World.

[41] Drosopoulos W.C., Prasad V.R. (2007). The active site residue Valine 867 in human telomerase reverse transcriptase influences nucleotide incorporation and fidelity. Nucleic Acids Res..

[42] Drosopoulos W.C., Prasad V.R. (2010). The telomerase-specific T motif is a restrictive determinant of repetitive reverse transcription by human telomerase. Mol. Cell. Biol..

[43] Latrick C.M., Cech T.R. (2010). POT1-TPP1 enhances telomerase processivity by slowing primer dissociation and aiding translocation. EMBO J..

[44] Kim S., Schroeder C.M., Xie X.S. (2010). Single-molecule study of DNA polymerization activity of HIV-1 reverse transcriptase on DNA templates. J. Mol. Biol..

[45] Gerard G.F., Potter R.J., Smith M.D., Rosenthal K., Dhariwal G., Lee J., Chatterjee D.K. (2002). The role of template-primer in protection of reverse transcriptase from thermal inactivation. Nucleic Acids Res..

[46] Parsons M.A., Sinden R.R., Izban M.G. (1998). Transcriptional properties of RNA polymerase II within triplet repeat-containing DNA from the human myotonic dystrophy and fragile X loci. J. Biol. Chem..

[47] Pham T.M., Tan K.W., Sakumura Y., Okumura K., Maki H., Akiyama M.T. (2013). A single-molecule approach to DNA replication in Escherichia coli cells demonstrated that DNA polymerase III is a major determinant of fork speed. Mol. Microbiol..

[48] Kennedy E.M., Gavegnano C., Nguyen L., Slater R., Lucas A., Fromentin E., Schinazi R.F., Kim B. (2010). Ribonucleoside triphosphates as substrate of human immunodeficiency virus type 1 reverse transcriptase in human macrophages. J. Biol. Chem..

[49] Zhao Y., Sfeir A.J., Zou Y., Buseman C.M., Chow T.T., Shay J.W., Wright W.E. (2009). Telomere extension occurs at most chromosome ends and is uncoupled from fill-in in human cancer cells. Cell.

[50] Zhao Y., Abreu E., Kim J., Stadler G., Eskiocak U., Terns M.P., Terns R.M., Shay J.W., Wright W.E. (2011). Processive and distributive extension of human telomeres by telomerase under homeostatic and nonequilibrium conditions. Mol. Cell.

