# Supplementary material for: Inventory of telomerase components in human cells reveals multiple subpopulations of hTR and hTERT
Source: Nucleic Acids Res. 2014 Jul 2;42(13):8565–77. doi: 10.1093/nar/gku560 (PMC4117779; doi:10.1093/nar/gku560)
Supplement: SUPPLEMENTARY DATA [file supp_42_13_8565__index.html]

Inventory of telomerase components in human cells reveals multiple subpopulations of hTR and hTERT — SUPPLEMENTARY DATA 

# Inventory of telomerase components in human cells reveals multiple subpopulations of hTR and hTERT

## SUPPLEMENTARY DATA

**Files in this Data Supplement:**

- SUPPLEMENTARY DATA
