# Supplementary material for: Inventory of telomerase components in human cells reveals multiple subpopulations of hTR and hTERT
Source: Nucleic Acids Res. 2014 Jul 2;42(13):8565–77. doi: 10.1093/nar/gku560 (PMC4117779; doi:10.1093/nar/gku560)
Supplement: SUPPLEMENTARY DATA [file supp_gku560_nar-00975-f-2014-File008.pdf]

SUPPLEMENTARY DATA

Figure S1. Validation of the hTR and hTERT RT-qPCR primers.
(a) Examination of the efficiencies of RT-qPCR primer pairs hTR 1-4. Each standard curve was generated by plotting the Ct values of the PCR reactions against the logarithm of the mass of Std hTR added (unit: fg) to base 10. The slopes of the standard curves are all close to -3.3, which is the theoretical value for a perfectly efficient primer pair. The function and R^2 value of each curve are shown. We also performed the template-titration experiment on cDNA prepared from HEK 293T total RNA, and found the four primer pairs were equally efficient in that context as well (data not shown). (b) Examination of the specificities of hTR RT-qPCR primer pairs 1-4. PCR products obtained with each primer pair, using cDNA prepared from HEK 293T total RNA as the template, were visualized by gel electrophoresis. Only a single band of the expected size was obtained with each primer pair tested. Lanes 1-4: RT-qPCR products obtained with primer pairs hTR 1-4; Lane 5: 50 bp DNA ladder (Promega). (c) Examination of the efficiencies of RT-qPCR primer pairs hTERT 1 and 2. Each standard curve was generated by plotting the Ct values of the PCR reactions against the logarithm of the mass of total cDNA added (unit: ng) to base 10. The slopes of the standard curves are both close to -3.3. The function and R^2 value of each curve are shown. (d) Examination of the specificities of hTERT RT-qPCR primer pairs 1 and 2. PCR products obtained with each primer pair, using cDNA prepared from HEK 293T total RNA as the template, were visualized by gel electrophoresis. Only a single band of the expected size was obtained with each primer pair tested. Lanes 1 and 4: RT-qPCR products obtained with primer pairs hTERT 1 and 2, respectively; Lane 2 and 3: 50 bp DNA ladder (Promega).

Figure S2. Analysis of hTERT and telomerase activity in the hTERT IP samples
HEK 293T and HeLa cell lysates were prepared in CHAPS lysis buffer as described in Materials and Methods. After the first round of hTERT IP, the flow through sample was used as the input sample for the second round of hTERT IP. (a) Upper panels: Telomerase activity in the original input, first round flow through (second round input) and second round flow through samples was examined by a direct assay (1 and 2, Duplicate repeats of experiment. +2 and +4, size markers made by extending the DNA primer by two or four nucleotides. LC, labelled unextended primer, serving as a loading control). Lower panels: Western blot with Abcam ab32020 was performed on these samples with β-actin as an internal control. (b) Summary of Abcam ab32020 western signals and telomerase activity levels present in the two rounds of flow through fractions, as normalized to input levels. Note that Abcam ab32020 western signal is not depleted although more than 70% of the telomerase activity is depleted after the two rounds of IP (compare 2nd round flow through with input).

Figure S3. Validation of methods used to prepare Std hTERT and quantification of hTERT protein levels in the whole cell lysate samples of HEK 293T and HeLa.
(a) FLAG-immunoprecipitated materials of *in vitro* transcription and translation in RRLs were analyzed by ^{35}S signal and by western blot. Lane 1: no hTERT plasmid template; Lane 2: no methionine; Lane 3-7:

titration of different amounts of cold methionine in the presence of constant, trace amounts of ^{35}S-methionine. The normalized reciprocal of incorporated ^{35}S-methionine counts per unit of hTERT was plotted against the amount of cold methionine added in Lane 4-7. As shown in Lane 1, if hTERT plasmid was omitted, little radioactivity was detected by SDS-PAGE; the same result was obtained from liquid scintillation counting (data not shown). Lanes 2-7 suggest that the RRLs contained pre-existing methionine. However, the pre-existing methionine concentration is negligible compared to the exogenously supplemented concentration, since the ensuing ratio of ^{35}S signal present in the Std hTERT protein (measured by SDS-PAGE) to the western blot signal of hTERT was inversely proportional to the amount of cold methionine added. (b) Assuming all the western signals detected by Abcam ab32020 in the cell lysate samples are from endogenous hTERT, hTERT protein levels in HEK 293T and HeLa cells were quantified by western blot. The number of hTERT molecules in the lysates was calculated by comparing the western blot signals obtained from these samples to that from a known number of Std hTERT molecules.

Figure S4. Examination of hTR and hTERT mRNA levels through cell cycle progression.
(a) Flow cytometry analysis of asynchronous or synchronous HeLa cell populations. Cells were synchronized at early S phase with double thymidine block and then released and analyzed after the indicated number of hours. (b) hTR levels and (c) hTERT mRNA levels in asynchronous and synchronous HeLa cell populations were examined by RT-qPCR, with 18S rRNA or U6 snRNA as internal control (n = 3).

Figure S5. Validation of methods used to quantify the specific activity of telomerase.
(a) Examination of how much of the radioactivity in the 18-mer LC sample resided in the oligonucleotide itself, instead of unincorporated nucleotides. The LC sample was run on a 10% polyacrylamide/7 M urea/1× TBE denaturing gel for different times as indicated. Quantification of the ^{32}P signal indicated that ~90% of the radioactivity is in the band of the 18-mer. (b) Time-course of telomerase activity. The telomerase assay was performed as described in Materials and Methods at the indicated temperature and time. Signals of extension products on the gel were normalized to that of the 18-mer LC (gel picture not shown). The amounts of extension products relative to that in the 30 °C, 0.5 h condition were plotted against the incubation time. (c) Telomerase assay of titrated immunopurified samples. The telomerase assay was performed as described in Materials and Methods at 37 °C for 2 h (1 and 2, Duplicate repeats of experiment. +2 and +4, size markers made by extending the DNA primer by two or four nucleotides. LC, labelled unextended primer, serving as a loading control). Lower panel: Signals of extension products on the gel were normalized to that of the 18-mer LC. The amounts of extension products relative to that of 1 μl super-telomerase elution were plotted in the bar graph (error bars: S. D., n = 2).

Figure S1

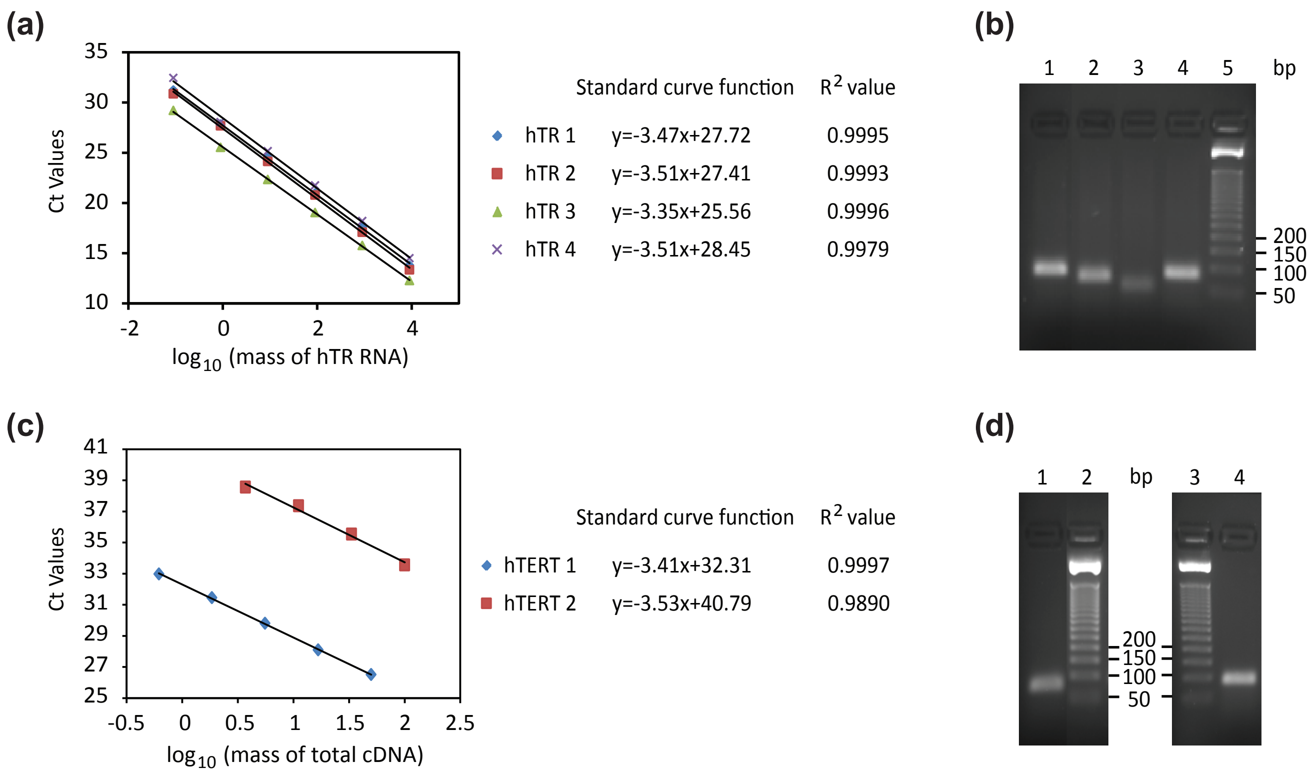

(a)
Ct Values
log10 (mass of hTR RNA)
Standard curve function
R2 value
hTR 1 y=-3.47x+27.72 0.9995
hTR 2 y=-3.51x+27.41 0.9993
hTR 3 y=-3.35x+25.56 0.9996
hTR 4 y=-3.51x+28.45 0.9979
(b)
1 2 3 4 5 bp
200
150
100
50
(c)
Ct Values
log10 (mass of total cDNA)
Standard curve function
R2 value
hTERT 1 y=-3.41x+32.31 0.9997
hTERT 2 y=-3.53x+40.79 0.9890
(d)
1 2 bp 3 4
200
150
100
50

Figure S2

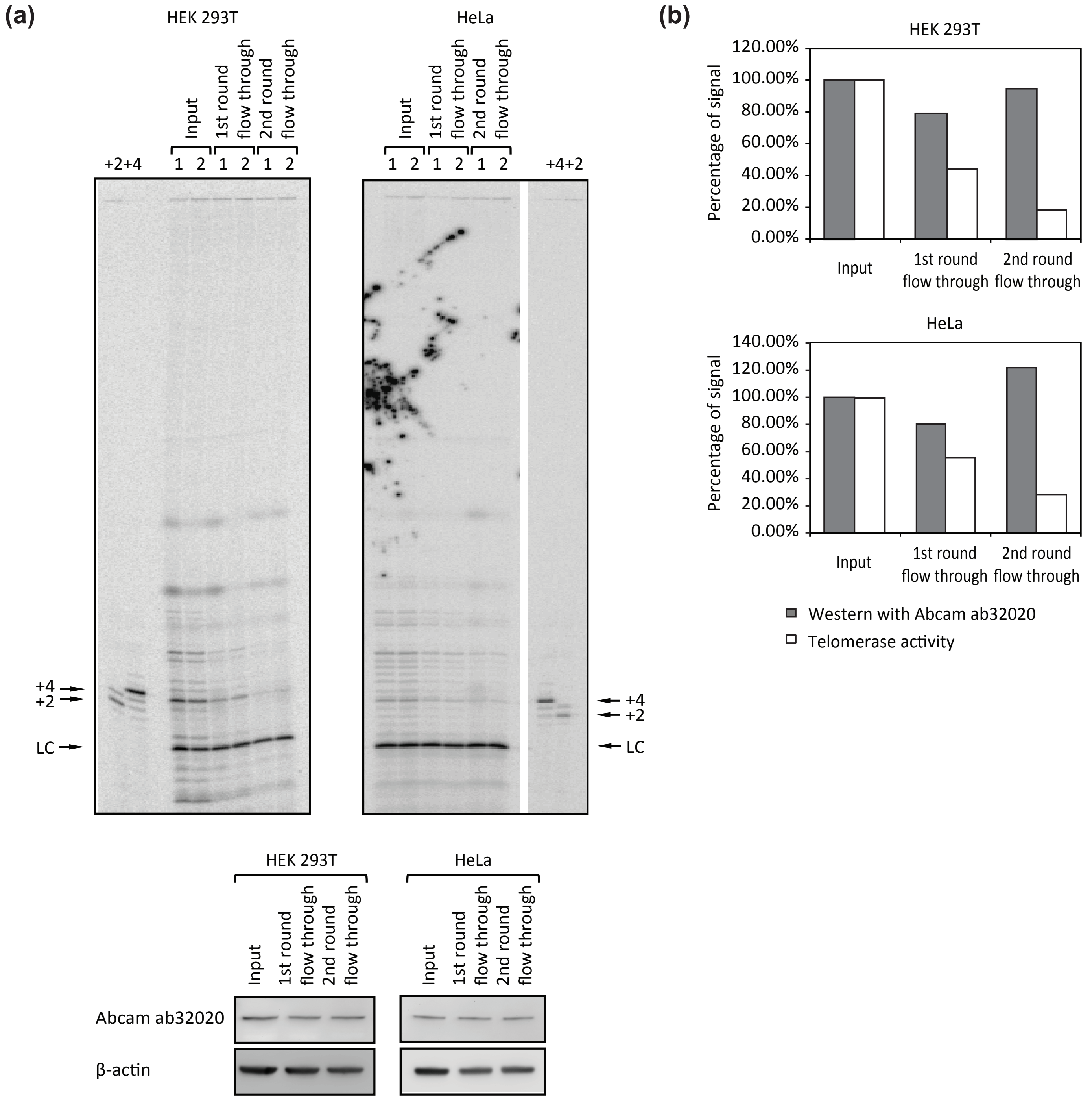
(a)
HEK 293T
Input
1st round flow through
2nd round flow through
1 2 1 2 1 2
+2+4
+4
+2
LC
HeLa
Input
1st round flow through
2nd round flow through
1 2 1 2 1 2
+4+2
+4
+2
LC
HEK 293T
HeLa
Input
1st round flow through
2nd round flow through
Abcam ab32020
β-actin
(b)
HEK 293T
Percentage of signal
120.00%
100.00%
80.00%
60.00%
40.00%
20.00%
0.00%
Input
1st round flow through
2nd round flow through
HeLa
Percentage of signal
140.00%
120.00%
100.00%
80.00%
60.00%
40.00%
20.00%
0.00%
Input
1st round flow through
2nd round flow through
Western with Abcam ab32020
Telomerase activity

Figure S3

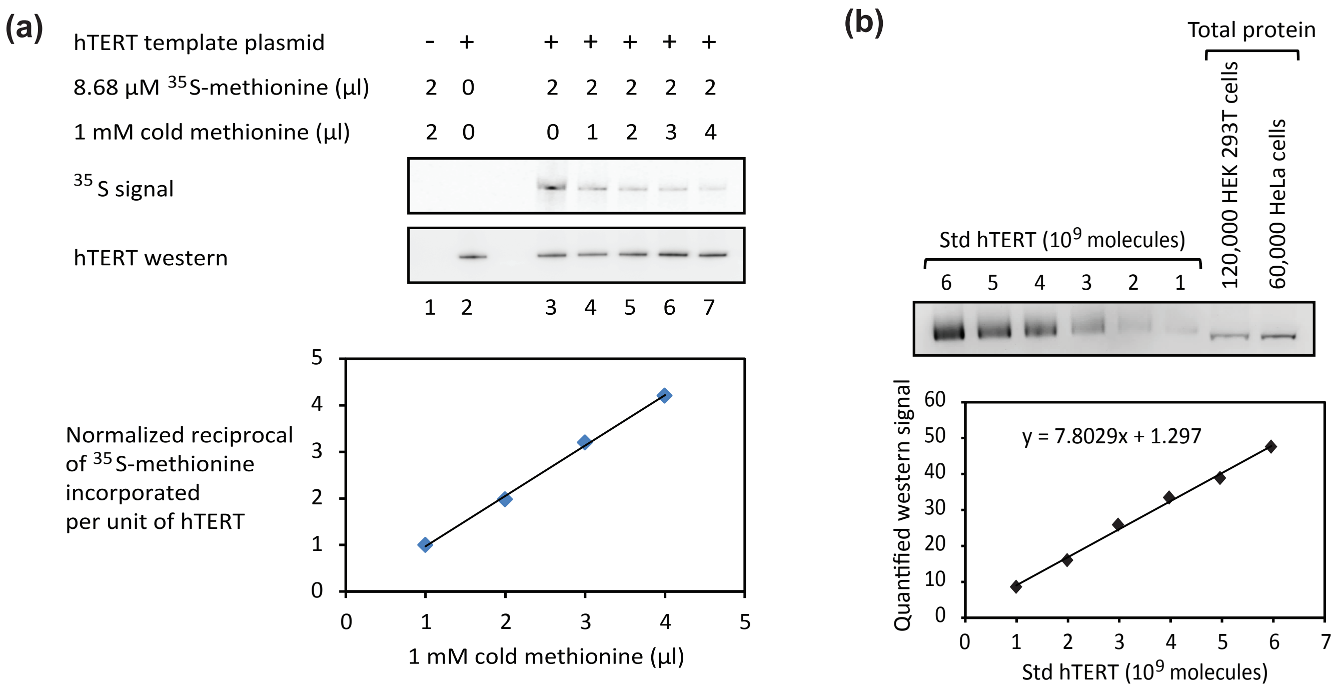

(a)
hTERT template plasmid
- + + + + + +
8.68 µM 35S-methionine (µl)
2 0 2 2 2 2 2
1 mM cold methionine (µl)
2 0 0 1 2 3 4
35 S signal
hTERT western
1 2 3 4 5 6 7
Normalized reciprocal of 35S-methionine incorporated per unit of hTERT
1 mM cold methionine (µl)
(b)
Total protein
120,000 HEK 293T cells
60,000 HeLa cells
Std hTERT (10^9 molecules)
6 5 4 3 2 1
Quantified western signal
y = 7.8029x + 1.297
Std hTERT (10^9 molecules)

Figure S4

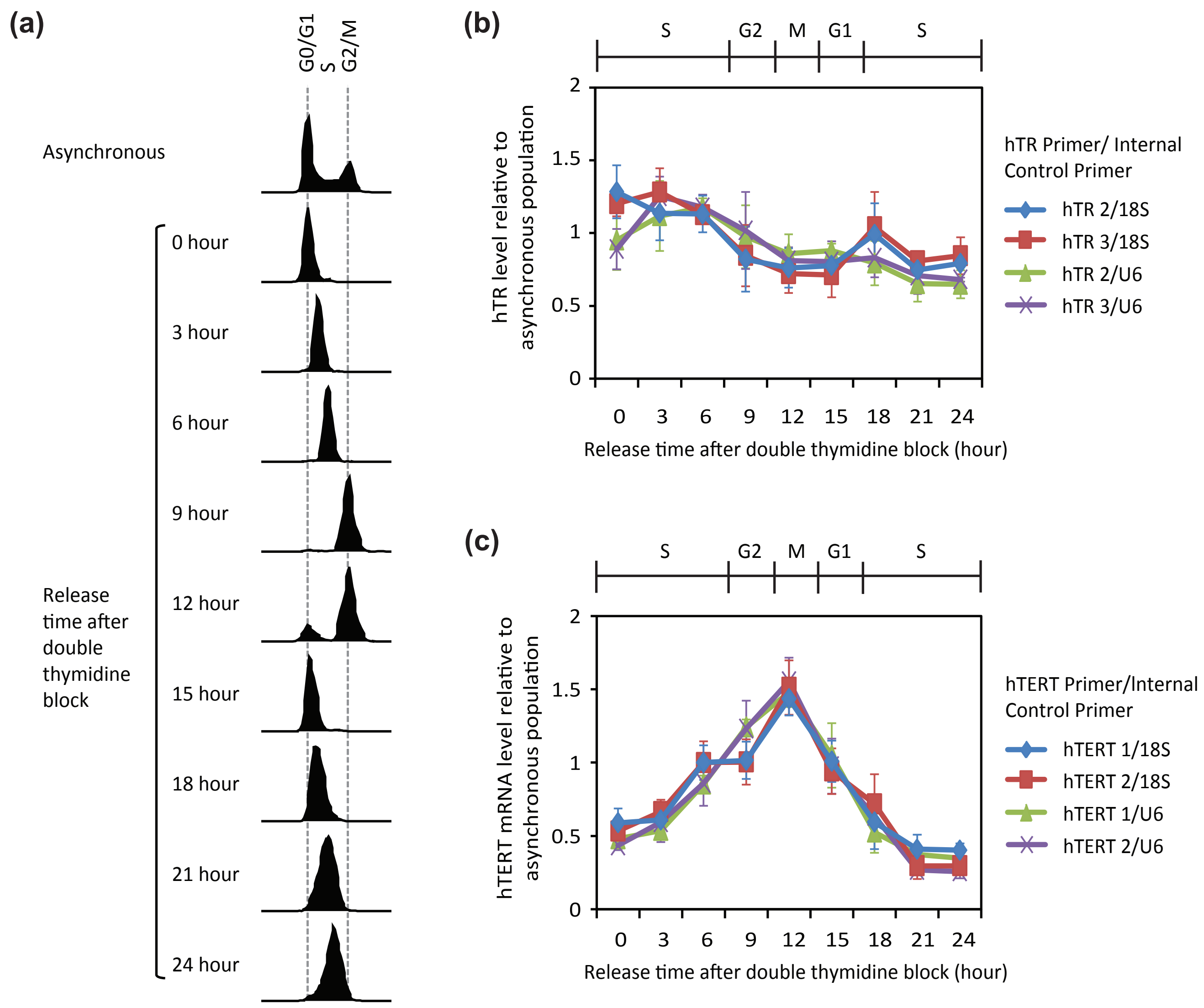
(a)
G0/G1
S
G2/M
Asynchronous
0 hour
3 hour
6 hour
9 hour
12 hour
15 hour
18 hour
21 hour
24 hour
Release time after double thymidine block
(b)
S
G2
M
G1
S
hTR level relative to asynchronous population
2
1.5
1
0.5
0
0
3
6
9
12
15
18
21
24
Release time after double thymidine block (hour)
hTR Primer/ Internal Control Primer
hTR 2/18S
hTR 3/18S
hTR 2/U6
hTR 3/U6
(c)
S
G2
M
G1
S
hTERT mRNA level relative to asynchronous population
2
1.5
1
0.5
0
0
3
6
9
12
15
18
21
24
Release time after double thymidine block (hour)
hTERT Primer/Internal Control Primer
hTERT 1/18S
hTERT 2/18S
hTERT 1/U6
hTERT 2/U6

Figure S5

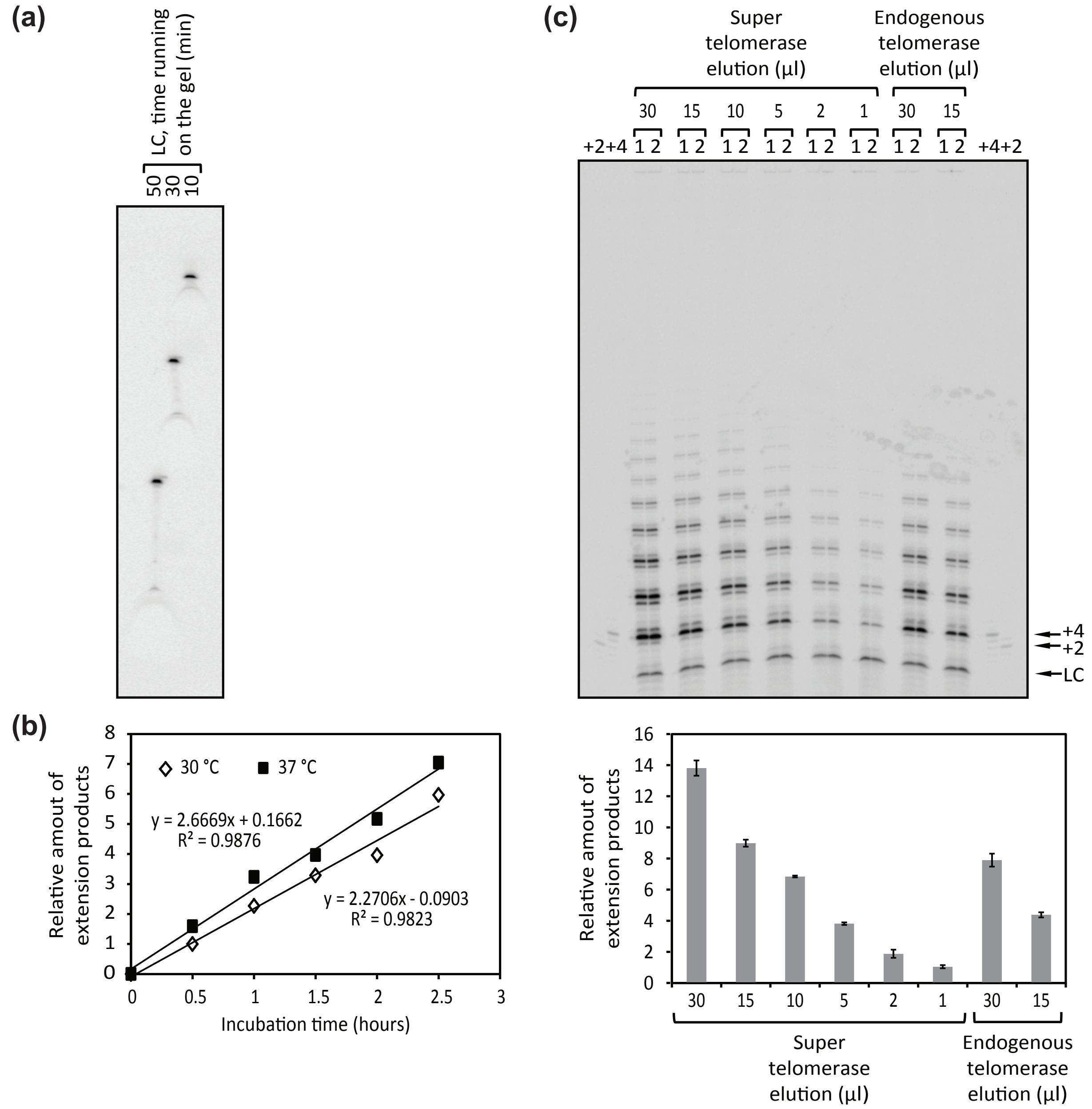

(a)
LC, time running on the gel (min)
50
30
10
(b)
30 °C
37 °C
y = 2.6669x + 0.1662
R² = 0.9876
y = 2.2706x - 0.0903
R² = 0.9823
Relative amout of extension products
Incubation time (hours)
0
0.5
1
1.5
2
2.5
3
1
2
3
4
5
6
7
8
(c)
Super telomerase elution (µl)
Endogenous telomerase elution (µl)
30
15
10
5
2
1
30
15
+2+4
1 2
+4+2
+4
+2
LC
Relative amout of extension products
0
2
4
6
8
10
12
14
16
30
15
10
5
2
1
30
15
Super telomerase elution (µl)
Endogenous telomerase elution (µl)

Table S1. Sequences of RT-qPCR primers.

Primer	Forward (5' to 3')	Reverse (5' to 3')
hTR 1	aagagttgggctctgtcagc	gactcgctccgttcctcttc
hTR 2	cgctgtttttctcgctgact	gctctagaatgaacggtggaa
hTR 3	aggggtggtggccatttt	cgcctacgcccttctcagt
hTR 4	gaagaggaacggagcgagtc	atgtgtgagccgagtcctg
hTERT 1	agcaccgtctgcgtgag	cagctcgacgacgtacacac
hTERT 2	catttcatcagcaagtttggaag	tttcaggatggagtagcagagg
GAPDH	acagcaacagggtggtggac	gaccattgctggggctggtg
U6	ctcgcttcggcagcaca	aacgcttcacgaatttgcgt
18S	cattcgaacgtctgccctat	gtttctcaggctccctctcc

Table S2. Sequences of northern blot probes.

Target gene	Probe (5' to 3')
hTR	gactcgctccgttcctcttc<br>gctctagaatgaacggtggaa<br>cctgaaaggcctgaacctc<br>cgcctacgcccttctcagt<br>atgtgtgagccgagtcctg
Human H1 RNA	ggagagtagtctgaattgggttatgagg<br>gctggccgtgagtctgttccaagctcc

Table S3. hTR copy number per cell [a] measured by different RT-qPCR primers (HEK293T hTR 3, n = 1; others, n = 2).

Primer	HEK293T	HeLa	VA13
hTR 1	650 ± 120	1040 ± 180	Undetectable
hTR 2	670 ± 60	1340 ± 70	Undetectable
hTR 3	1260	1840 ± 100	Undetectable
hTR 4	930 ± 210	1550 ± 120	Undetectable

[a] All values are expressed as mean ± S. D..

Detailed calculation steps for specific quantification analysis

1. Quantification of endogenous hTR levels through RT-qPCR

Take the quantification of the hTR level in HEK 293T cells using RT-qPCR with primer pair hTR 1 (Figure 1a) as an example. Total RNA extracted from ~2140 HEK 293T cells was mixed with 0, 1, 2, 3, 4, 5 or 6 million Std hTR molecules. RT-qPCR was performed on cDNA samples prepared from these mixtures with the primer pair hTR 1 as well as an internal control primer pair for GAPDH. Based on the Ct values, the ratio of Std hTR/endogenous hTR in each sample was calculated with the following formula:

In sample n, to which n million Std hTR molecules were added,

$$\frac{\text{Std hTR}}{\text{Endogenous hTR}} = 2^{[(\text{Ct0hTR 1}-\text{Ct0GAPDH})-(\text{CtnhTR 1}-\text{CtnGAPDH})]} - 1.$$

Ct0hTR 1: Ct value of the PCR reaction with the primer pair hTR 1 and cDNA from the sample 0;
Ct0GAPDH: Ct value of the PCR reaction with the primer pair GAPDH and cDNA from the sample 0;
CtnhTR 1: Ct value of the PCR reaction with the primer pair hTR 1 and cDNA from the sample n;
CtnGAPDH: Ct value of the PCR reaction with the primer pair GAPDH and cDNA from the sample n.

Then the calculated ratio of Std hTR to endogenous hTR was plotted against the amount of Std hTR added to make a standard curve, as shown in Figure 1a. In this particular case, the function of the standard curve is y = 0.6336x + 0.0051. When y = 1, x is calculated to be 1.57, which means the amount of endogenous hTR molecules present in each sample is ~1.57 million. Dividing this number by the number of cells from which the total RNA was extracted (2140 in this case), the average hTR copy number per cell is estimated to be ~730 in this experiment.

2. Quantification of Std hTERT protein levels based on ^{35}S signal

As described in Materials and Methods, 10 μl 1.0 mM L-methionine and 10 μl ^{35}S-L-methionine (1 mCi in 98 μl, 1175 Ci/mmol, which equals 8.68 μM, PerkinElmer) were added to the 500 μl reaction system to prepare Std hTERT protein. ^{35}S signals in the original reaction system and in the beads slurry after immunoprecipitation and washing were measured by liquid scintillation counting. Then the Std hTERT protein on the beads was examined by SDS-PAGE to determine the percentage of the radioactivity present in the protein band.

By liquid scintillation counting, we measured the ^{35}S signal in the original reaction system and in the beads slurry to be 464373 cpm/μl and 14985 cpm/μl, respectively. Therefore, the ratio of methionine present in each μl of the beads slurry to that added to the reaction system at the beginning was calculated:

$$\frac{14985\ \text{cpm/μl} \times 1\ \text{μl}}{464373\ \text{cpm/μl} \times 500\ \text{μl}} \approx 6.5\times10^{-5}.$$

The total amount of methionine added to the system is calculated by adding the amount of cold methionine and the amount of hot methionine together.

$$\text{Amount of cold methionine} = 1.0\ \text{mM} \times 10\ \mu\text{l} = 1.0 \times 10^{-8}\ \text{mol};$$
$$\text{Amount of hot methionine} = 8.68\ \mu\text{M} \times 10\ \mu\text{l} = 8.68\times10^{-11}\ \text{mol};$$
$$\text{Total amount of methionine} = 1.0 \times 10^{-8}\ \text{mol} + 8.68\times10^{-11}\ \text{mol} = 1.0 \times 10^{-8}\ \text{mol}.$$

Then the amount of methionine present in each μl of the beads slurry should be:

$$(1.0 \times 10^{-8}\ \text{mol}) \times (6.5\times10^{-5}) \times (6.02 \times10^{23}\ \text{molecules/mol}) = 3.9\times10^{11}\ \text{molecules}.$$

As SDS-PAGE analysis indicates ~93% of the ^{35}S signal in the beads slurry is present in the protein band (Figure 3a), and there are 13 methionine amino acids in each 3×FLAG-hTERT protein, the molecule number of hTERT protein present in each μl of the beads slurry should be:

$$(3.9\times10^{11}) \times 93\%/13 = 2.8\times10^{10}.$$

3. Quantification of the specific activity of telomerase

As described in Materials and Methods, in each direct enzyme assay, 30 μl telomerase was added to a 20 μl assay buffer containing 25 μM dGTP and 3 μl [α-^{32}P]-dGTP (10 μCi/μl, 3000 Ci/mmol, Perkin-Elmer). The reaction was carried out for 2 h at 37 °C, and then quenched by adding 250 μl 3.6 M NH_4OAc with a 5'-^{32}P-labeled 18-mer as loading control.

First, we determined the ^{32}P signal present in the band of the 18-mer LC. By liquid scintillation counting, we measured that 3063 cpm radioactivity was present in the LC sample we added to each reaction system. Examination of the LC sample on the 10% polyacrylamide/7 M urea/1× TBE denaturing gel (Figure S5a) indicated that ~90% of the ^{32}P signal was in the band of the 18-mer, which corresponded to 2746 cpm. We then determined the ratio of the ^{32}P signal present in the extension products to the ^{32}P signal present in the 18-mer band on the polyacrylamide gel. Taking replicate 1 of the endogenous telomerase elution in Figure 5b as an example, the ratio was ~1.7, so the ^{32}P signal present in the extension products was calculated:

$$2746\ \text{cpm} \times 1.7 = 4668\ \text{cpm}.$$

The total ^{32}P signal in the [α-^{32}P]-dGTP added to the assay was measured by liquid scintillation counting, giving ~8.9×10^{7} cpm. Thus, the ratio of dGTP incorporated in the extension products to the total dGTP added should be:

$$4668\ \text{cpm} / 8.9\times10^{7}\ \text{cpm} = 5.2\times10^{-5}.$$

The total amount of dGTP added to the system is calculated by adding the amount of cold dGTP and the amount of hot dGTP, as follows:

Amount of cold dGTP = 25 μM × 20 μl = 5.0×10^{-10} mol;

Amount of hot dGTP = (3.0 μl × 10 μCi/μl)/(3000 Ci/mmol) = 1.0×10^{-11} mol;

Total amount of dGTP = 5.0×10^{-10} mol + 1.0×10^{-11} mol = 5.1×10^{-10} mol.

Then the amount of dGTP incorporated in the extension products was then calculated:

$$5.1\times10^{-10} \text{ mol} \times (5.2\times10^{-5}) = 2.7\times10^{-14} \text{ mol.}$$

As the sequence of the extension products is the repetition of "GGGTTA", only half of which is G, the amount of total deoxyribonucleotides incorporated equals:

$$2.7\times10^{-14} \text{ mol} \times 2 = 5.4\times10^{-14} \text{ mol.}$$

By northern blot, the amount of hTR in the elution sample added to this direct assay is 2.29×10^{-17} mol (Figure 5a), which is also the amount of telomerase monomers added to this assay. Therefore, the specific activity of telomerase quantified by this experiment is:

$$5.4\times10^{-14} \text{ mol nucleotides} / (2.29\times10^{-17} \text{ mol telomerase monomers} \times 120 \text{ min})$$

$$= 19.3 \text{ nucleotides incorporated per telomerase monomer per min.}$$
